# Identification of prognostic biomarkers associated with the occurrence of portal vein tumor thrombus in hepatocellular carcinoma

**DOI:** 10.18632/aging.202876

**Published:** 2021-04-20

**Authors:** Tong Lin, Zhimei Lin, Peipei Mai, E Zhang, Lisheng Peng

**Affiliations:** 1 The Fourth Clinical Medical School, Guangzhou University of Chinese Medicine, Shenzhen, China; 2 Department of Science and Education, Shenzhen Hospital of Traditional Chinese Medicine, Shenzhen, China

**Keywords:** hepatocellular carcinoma, portal vein tumor thrombus, bioinformatics, prognosis, immune infiltration

## Abstract

The occurrence of portal vein tumor thrombus (PVTT) is strongly correlated to the staging and poor prognosis of hepatocellular carcinoma (HCC) patients. However, the mechanisms of PVTT formation remain unclear. This study aimed to investigate differentially expressed genes (DEGs) between primary tumor (PT) and PVTT tissues and comprehensively explored the underlying mechanisms of PVTT formation. The DEGs between PT and paired PVTT tissues were analyzed using transcriptional data from the Gene Expression Omnibus (GEO) database. The expression, clinical relevance, prognostic significance, genetic alternations, DNA methylation, correlations with immune infiltration, co-expression correlations, and functional enrichment analysis of the DEGs were explored using multiple databases. As result, 12 DEGs were commonly down-expressed in PVTT compared with PT tissues among three datasets. The expression of *DCN*, *CCL21*, *IGJ*, *CXCL14*, *FCN3*, *LAMA2*, and *NPY1R* was progressively decreased from normal liver, PT, to PVTT tissues, whose up-expression associated with favorable survivals of HCC patients. The genetic alternations and DNA methylation of the DEGs frequently occurred, and several methylated CpG sites of the DEGs significantly correlated with outcomes of HCC patients. The immune infiltration in the tumor microenvironment of HCC was correlated with the expression level of the DEGs. Besides, the DEGs and their co-expressive genes participated in the biological processes of extracellular matrix (ECM) organization and focal adhesion. In summary, this study indicated the dysregulation of ECM and focal adhesion might contribute to the formation of PVTT. And the above seven genes might serve as potential biomarkers of PVTT occurrence and prognosis of HCC patients.

## INTRODUCTION

Hepatocellular carcinoma (HCC) is the sixth most frequently diagnosed and the fourth deadliest cancer worldwide [1]. Blamed on the occult early symptoms, a majority of HCC patients are diagnosed at advanced stages with metastasis [2]. Portal vein tumor thrombosis (PVTT) is a dominant form of intrahepatic vessel metastasis which occurs in 10 - 40% of HCC patients at first diagnosis [3, 4]. The formation of PVTT can induce intrahepatic metastasis, deteriorated hepatic function, poor tolerance to treatment, and a series of complications following portal hypertension [5]. The median overall survival (OS) of HCC patients with PVTT is merely 2.7 - 4.0 months if without effective treatments [6].

Characterizing molecular biomarkers between primary tumor (PT) and PVTT is necessary for the early diagnosis and treatment of HCC patients with PVTT. However, the knowledge about PVTT formation is limited so far [7, 8]. The similar transcriptional alternations between PT and PVTT implied PVTT might originate from PT by metastasis [9–11]. Contradictory, some alterations were identified between PT and paired PVTT in some HCC patients, which indicated PVTT might have different origins from PT, and high inter-patient heterogeneity exists [12, 13]. Ye et al. found osteopontin was over-expressed in metastatic HCC which could regulate invasion and metastasis of HCC cells [9]. Zhang et al. observed dysregulated genes involved with extracellular matrix (ECM)-receptor interaction might relate with the venous metastasis of HCC [14]. Wang et al. identified 20 recurrently and progressively differentially expressed genes (DEGs) from matched adjacent normal, PT, and PVTT samples. These genes participated in focal adhesion and xenobiotics metabolism, and many of them could regulate the invasion of HCC cells [13]. Besides, genomic variations [12], non-coding RNAs [10, 15], DNA methylation [16], cancer stem cells [17], along with the immune cells [18] and vascular endothelial cells [19] in the tumor microenvironment (TME) have been reported to contribute to the development of PVTT.

To learn the mechanisms of the formation of PVTT, this study investigated the DEGs between PT and PVTT tissues using the transcriptional profiles from the Gene Expression Omnibus (GEO) database. The mRNA expression, clinical relevance, prognostic significance, genetic alternations, DNA methylation, correlations with the immune infiltration, and biological functions of the DEGs in HCC were comprehensively explored applying integrated bioinformatic analyses. Our study might throw lights on the molecular mechanisms of PVTT formation and inspire novel insights for further researches and therapeutic strategies. The workflow of this study is displayed in Supplementary Figure 1.

## RESULTS

### Identification of the DEGs between PT and PVTT tissues

**Figure 1:**
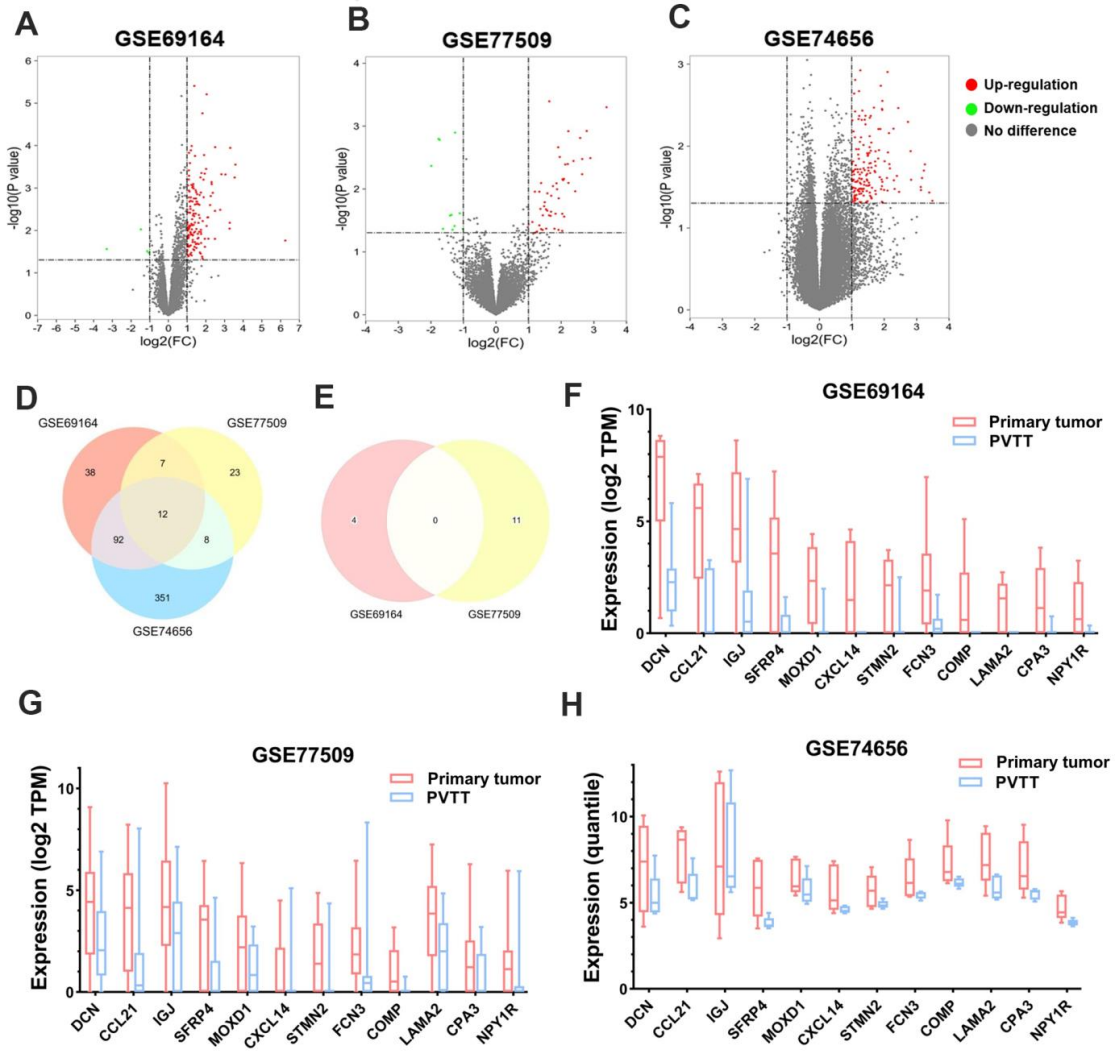
**Identification of the DEGs between PT and PVTT tissues.** Volcano plots showing the identification of the DEGs in (**A**) GSE69164, (**B**) GSE77509, and (**C**) GSE74656 datasets with the screening criteria of |log_2_(FC)| > 1 and *P* < 0.05. Dots in red or green represent upregulated or downregulated DEGs in PT compared with PVTT tissues, dots in grey represent genes without significant expressional differences. Venn diagrams showing the intersections of (**D**) upregulated and (**E**) downregulated DEGs among the three datasets. Since no downregulated DEGs was identified in dataset GSE74656, the Venn diagram for it was not drawn. The expression of the 12 overlapping upregulated DEGs in PT and PVTT tissues in datasets (**F**) GSE69164, (**G**) GSE77509, and (**H**) GSE74656. DEGs, differentially expressed genes; PT, primary tumor; PVTT, portal vein tumor thrombus; FC, fold change.

To begin with, the DEGs were screened in each of the three GEO datasets. It turned out that 149, 50, and 463 DEGs were significantly upregulated (log_2_FC > 1, *P* < 0.05), while 4, 11, and zero DEGs were downregulated (log_2_FC < -1, *P* < 0.05) in PT compared with paired PVTT tissues in GSE69164, GSE77509, and GSE74656 datasets, respectively (Figure 1A–1C). Then, twelve upregulated DEGs, *DCN*, *CCL21*, *IGJ* (*JCHAIN*), *SFRP4*, *MOXD1*, *CXCL14*, *STMN2*, *FCN3*, *COMP*, *LAMA2*, *CPA3*, and *NPY1R* were found intersected among the three datasets (Figure 1D); while no overlapping downregulated DEGs was observed between GSE69164 and GSE77509 datasets (Figure 1E). The 12 DEGs that showed lower expression levels in PVTT than PT tissues might involve in the formation of PVTT, so they were further investigated in this study. The expression profiles of the 12 DEGs are shown in Figure 1F–1H and Table 1.

**Table 1:** Expression of the 12 DEGs in primary tumor compared with paired PVTT tissues in three GEO datasets.

**Gene symbol**	**Protein name**	**GSE69164**			**GSE77509**			**GSE74656**	
		**Log_2_FC**	***P* value**		**Log_2_FC**	***P* value**		**Log_2_FC**	***P* value**
*DCN*	Decorin	3.591	2.82E-04		1.542	4.29E-02		3.393	3.70E-02
*CCL21*	C-C motif chemokine 21	3.055	4.82E-04		2.897	3.24E-03		2.178	1.88E-02
*IGJ*	Immunoglobulin J chain	2.776	3.29E-03		2.035	2.49E-02		1.437	4.05E-02
*SFRP4*	Secreted frizzled-related protein 4	2.321	8.12E-04		1.927	1.41E-02		2.736	5.08E-03
*MOXD1*	DBH-like monooxygenase protein 1	1.912	1.68E-04		1.696	1.29E-02		1.078	7.01E-03
*CXCL14*	C-X-C motif chemokine 14	1.771	4.45E-04		2.224	8.05E-03		2.393	2.32E-02
*STMN2*	Stathmin-2	1.740	1.29E-03		3.396	5.04E-04		1.732	1.16E-02
*FCN3*	Ficolin-3	1.644	1.09E-02		1.386	4.36E-02		1.696	5.01E-02
*COMP*	Cartilage oligomeric matrix protein	1.478	1.77E-03		2.645	5.85E-03		1.382	2.98E-02
*LAMA2*	Laminin subunit alpha-2	1.389	3.95E-06		1.578	1.18E-02		1.951	4.88E-02
*CPA3*	Carboxypeptidase A4	1.107	7.80E-03		1.436	2.64E-02		1.601	4.22E-02
*NPY1R*	Neuropeptide Y receptor type 1	1.050	8.74E-04		1.574	2.22E-02		1.942	3.62E-03

### Expression of the DEGs in HCC and their clinical relevance

**Figure 2:**
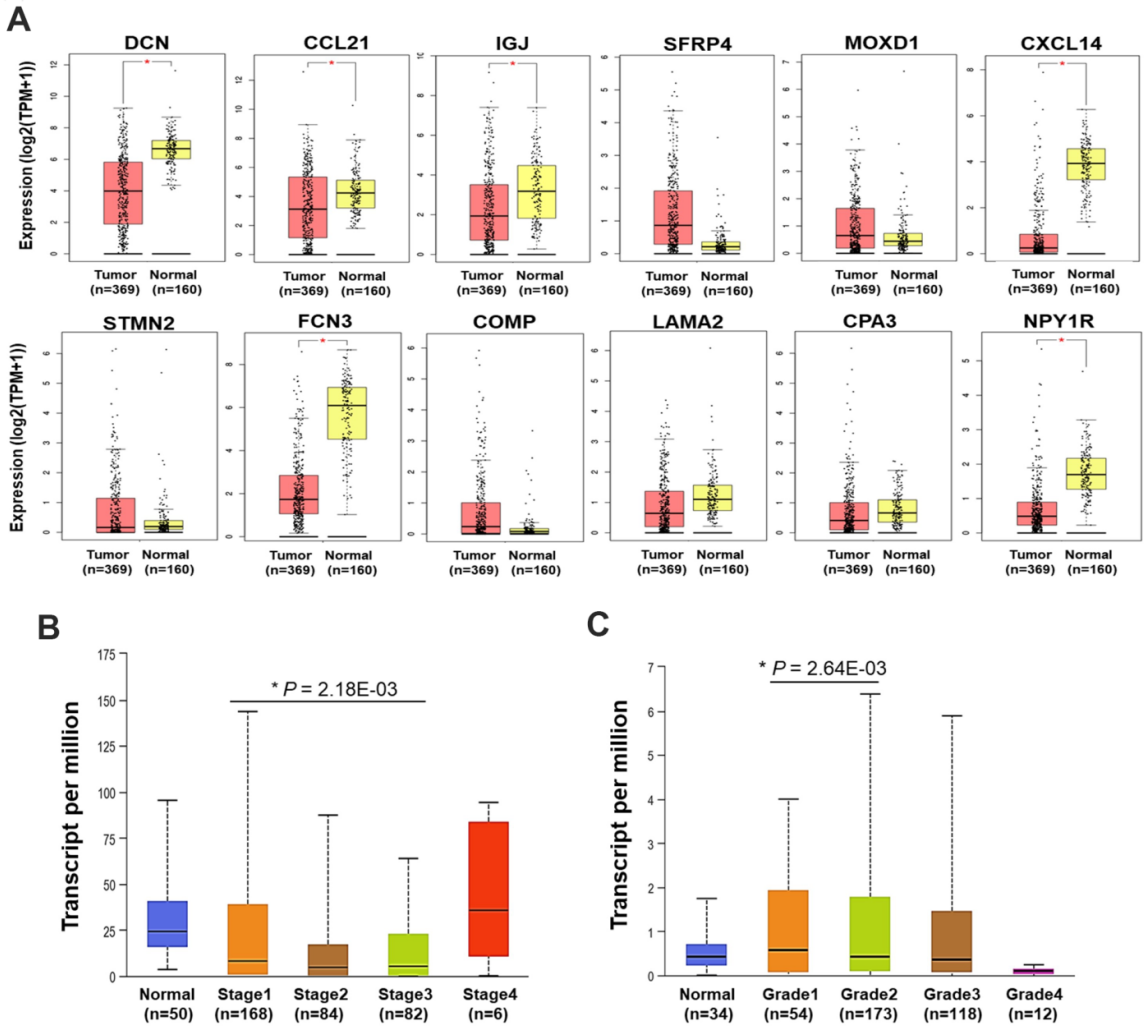
**Expression and clinical relevance of the DEGs.** (**A**) The expression of the 12 DEGs in HCC and normal liver samples (GEPIA) (*P < 0.05). (**B**) The expression of *CCL21* in HCC and normal liver samples by different stages. (**C**) The expression of *MOXD1* in HCC and normal liver samples by different grades. TPM, transcript per million.

Following, the expression of the 12 DEGs in HCC tumors and normal liver samples was validated using TCGA data via GEPIA platform. It showed that the mRNA expression of *DCN*, *CCL21*, *IGJ*, *CXCL14*, *FCN3*, and *NPY1R* was significantly decreased in HCC compared with normal liver samples (Figure 2A).

Then, correlations between the expression of the 12 DEGs with the clinical characteristics were analyzed using UALCAN. In general, we found *DCN*, *IGJ*, *CXCL14*, *FCN3*, and *NPY1R* were significantly down-expressed, while *SFRP4* and *COMP* were up-expressed in HCC samples with distinct genders, ages, stages, and grades, compared with normal liver samples (*P* < 0.05). Different from the findings in GEPIA platform, the down-expression of *CCL21* in HCC was not statistically significant here, compared with normal liver samples (Supplementary Figure 2A–2D). Furthermore, the expression level of *CCL21* in Stage-I HCC was higher than that in Stage-III HCC (*P* < 0.05) (Figure 2B). *MOXD1* was expressed higher in Grade 1 tumor than that in Grade 2 tumor (*P* < 0.05) (Figure 2C).

### Prognostic significance of the DEGs in all HCC patients

**Figure 3:**
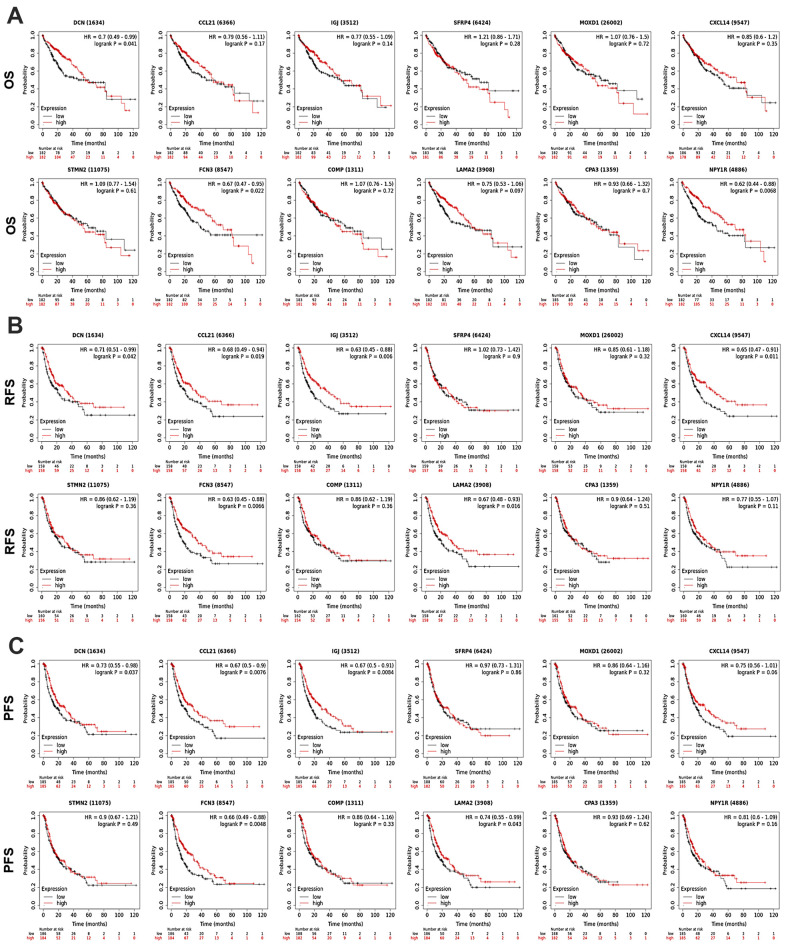
**Prognostic significance of the DEGs in HCC patients (KM Plotter).** The survival curves showed the associations between the expression of the 12 DEGs with (**A**) OS, (**B**) RFS, and (**C**) PFS of HCC patients. OS, overall survival; RFS, relapse free survival; PFS, progression free survival; HR, hazard ratio.

To discover the prognostic values of the 12 DEGs in HCC patients, survival analyses were performed using KM Plotter. As shown in Figure 3, high-expression of *DCN* (OS: HR = 0.7, *P* = 0.041; RFS: HR = 0.71, *P* = 0.042; PFS: HR = 0.73, *P* = 0.037) and *FCN3* (OS: HR = 0.67, *P* = 0.022; RFS: HR = 0.63, *P* = 0.0066; PFS: HR = 0.66, *P* = 0.0048) was associated with longer OS, RFS, and PFS of all HCC patients. Upregulation of *CCL21* (RFS: HR = 0.69, *P* = 0.019; PFS: HR = 0.67, *P* = 0.0076), *IGJ* (RFS: HR = 0.63, *P* = 0.006; PFS: HR = 0.67, *P* = 0.0084), and *LAMA2* (RFS: HR = 0.67, *P* = 0.016; PFS: HR = 0.74, *P* = 0.043) linked with better RFS and PFS of all HCC patients. High-expression of *NPY1R* and *CXCL14* associated with favorable OS (HR = 0.62, *P* = 0.0068) and RFS (HR = 0.65, *P* = 0.011) of all HCC patients, respectively. Except for the above, the expression of *SFRP4*, *COMP*, *MOXD1*, *STMN2*, and *CPA3* presented no significant association with HCC patients’ prognosis.

### Prognostic values of the DEGs in HCC patients with diverse clinical characteristics

Subsequently, associations between the DEGs’ expression with OS and PFS of HCC patients with diverse clinical characteristics were further assessed. Male HCC patients with higher expression of *FCN3* (HR = 0.60, *P* = 0.026) and *NPY1R* (HR = 0.53, *P* = 0.005) might have better OS; those patients with higher expression of *DCN* (HR = 0.62, *P* = 0.009), *CCL21* (HR = 0.63, *P* = 0.012), *IGJ* (HR = 0.66, *P* = 0.023), *FCN3* (HR = 0.67, *P* = 0.031), and *LAMA2* (HR = 0.63, *P* = 0.012) might have better PFS. Elevated expression of *IGJ* (OS: HR = 0.61, *P* = 0.014; PFS: HR = 0.63, *P* = 0.021) and *FCN3* (OS: HR = 0.61, *P* = 0.044; PFS: HR = 0.66, *P* = 0.037) linked with favorable OS and PFS; and high-expression of *DCN* (HR = 0.61, *P* = 0.014) and *LAMA2* (HR = 0.63, *P* = 0.02) associated with better PFS of HCC patients without a family history of cancer (Supplementary Tables 1, 2).

In terms of etiological factors, high-expression of *DCN* (OS: HR = 0.31, *P* = 0.028; PFS: HR = 0.32, *P* = 0.002), *IGJ* (OS: HR = 0.61, *P* = 0.014; PFS: HR =0.43, *P* = 0.018), *CXCL14* (OS: HR = 0.21, *P* = 0.007; PFS: HR =0.44, *P* = 0.019), and *LAMA2* (OS: HR = 0.31, *P* = 0.029; PFS: HR = 0.36, *P* = 0.004) associated with both better OS and PFS of HCC patients with a history of hepatitis B; and upregulation of *CCL21* (HR = 0.47, *P* = 0.031) and *FCN3* (HR = 0.49, *P* = 0.044) associated with better PFS of these patients. As for HCC patients with alcohol consumption, high-expressed *FCN3* (HR = 0.21, *P* = 0.0003) and *NPY1R* (HR = 0.47, *P* = 0.04) indicated better OS; *LAMA2* (HR = 0.53, *P* = 0.032) suggested better PFS of these patients.

When it comes to pathological stages and grades, up-expression of *IGJ* (HR = 0.47, *P* = 0.005), *STMN2* (HR = 0.51, *P* = 0.013), *COMP* (HR = 0.58, *P* = 0.044), and *NPY1R* (HR = 0.52, *P* = 0.014) related with favorable PFS of patients in advanced stages (Stage III-IV). *NPY1R* upregulation also indicated better OS (HR = 0.39, *P* = 0.002) of advanced-stage patients and better OS (HR = 0.51, *P* = 0.032) of patients with Grade 3 tumor. Besides, *FCN3* overexpression linked with both favorable OS (HR = 0.58, *P* = 0.037) and PFS (HR = 0.64, *P* = 0.048) of patients with Grade 2 tumor.

### Alternations of the DEGs in HCC patients

**Figure 4:**
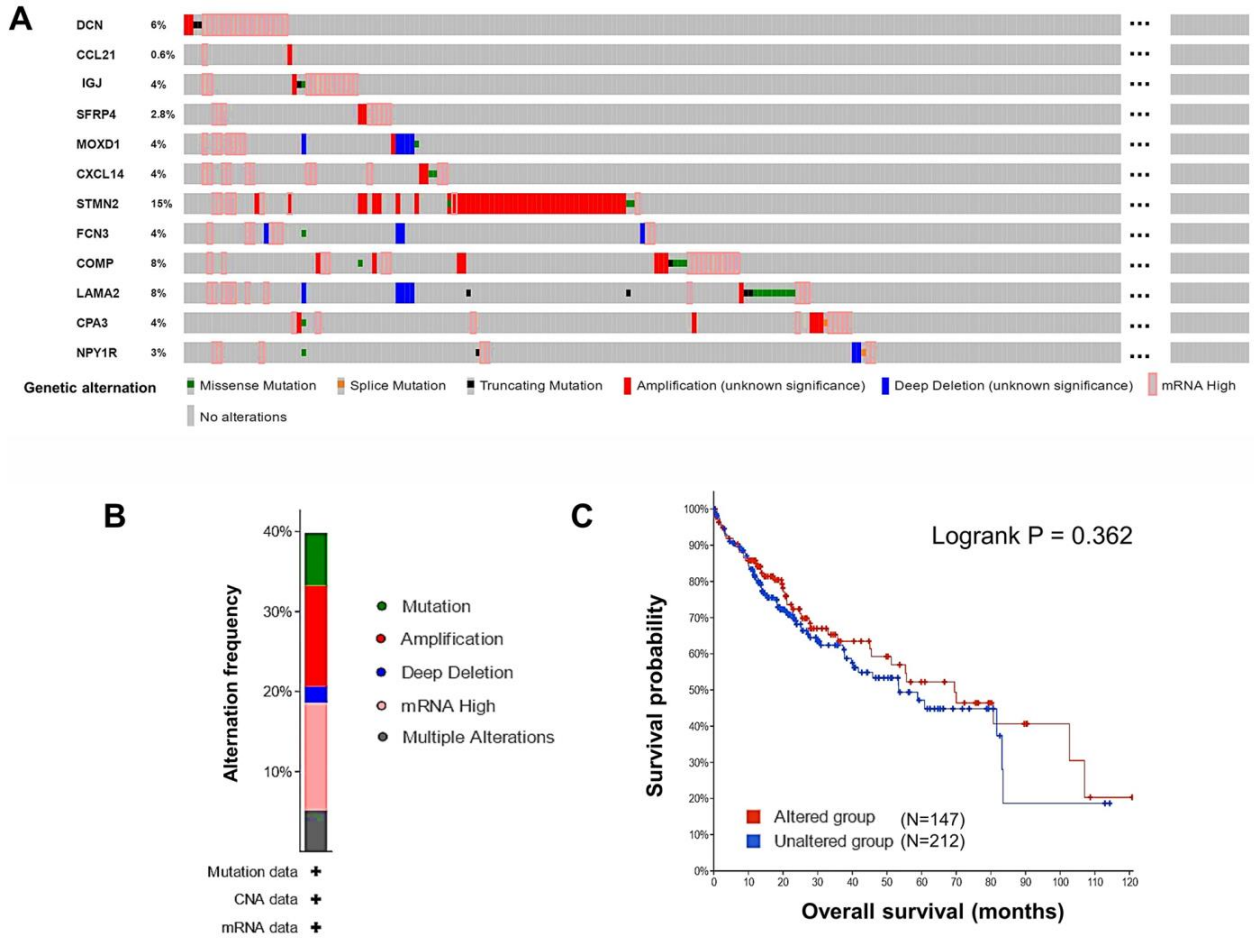
**Genetic alternations of the DEGs in HCC patients (cBioPortal).** (**A**) The overview of the genetic alternations occurring in the 12 DEGs in HCC patients from “TCGA, Firehose Legacy” dataset. (**B**) The summary graph of alternation frequency of the 12 DEGs in HCC patients. (**C**) The effect of the overall genetic alternations of the DEGs on OS of HCC patients.

Next, alternations of the 12 DEGs in HCC patients were analyzed using cBioPortal. Overall, six kinds of alternations, including missense mutation, slice mutation, truncating mutation, amplification, deep deletion, and mRNA overexpression of the DEGs were observed in a total of 147 out of 360 (41%) HCC samples (Figure 4A). The most frequent alternation was mRNA overexpression which occurred in 47 (13.47%) cases (Figure 4B), and *STMN2* was the most frequently (15%) altered gene. In Figure 4A, we could see some samples with alterations in one gene tended to have alterations in other genes. This observation could be supported by the results of co-occurrence analysis, that alterations in 12 pairs of genes significantly (*P* < 0.01) co-occurred in the same samples (Supplementary Table 3). The alternations of the DEGs indicated their potential participation in the development of HCC. However, the overall genetic alterations in the DEGs were not significantly related to OS of HCC patients (Figure 4C).

### Prognostic value of DNA methylation of the DEGs in HCC

**Figure 5:**
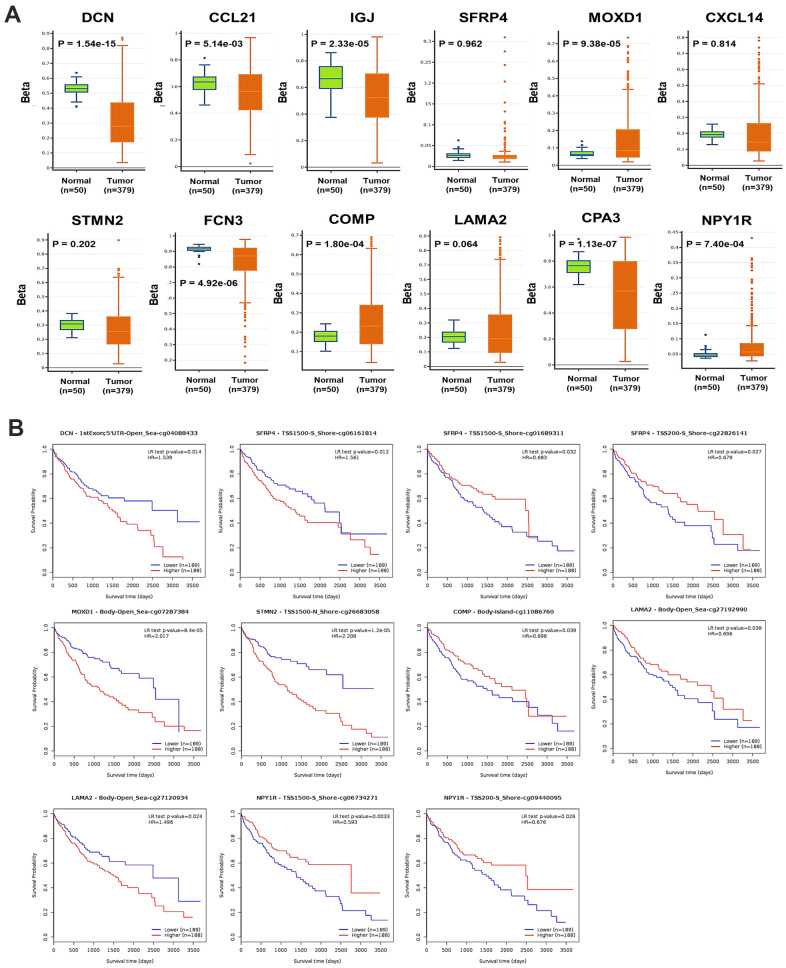
**DNA methylation of the DEGs in HCC.** (**A**) The global DNA methylation level of the 12 DEGs in HCC and normal liver samples (DNMIVD). (**B**) The associations between the methylation level of CpG sites of the DEGs with the OS of HCC patients (MethSurv).

Generally, the global DNA methylation of *DCN*, *CCL21*, *IGJ*, *FCN3*, and *CPA3* was significantly decreased, while that of *MOXD1* and *NPY1R* was significantly increased in HCC, compared with normal liver samples (Figure 5A). In detail, 9 CpG sites of *DCN*, 1 CpG site of *CCL12*, 2 CpG sites of *IGJ*, 16 CpG sites of *SFRP4*, 13 CpG sites of *MOXD1*, 11 CpG sites of *CXCL14*, 18 CpG sites of *STMN2*, 7 CpG sites of *FCN3*, 22 CpG sites of *COMP*, 33 CpG sites of *LAMA2*, 4 CpG sites of *CPA3*, and 18 CpG sites of *NPY1R* were found significantly differently methylated in HCC, compared with normal samples (Supplementary Table 4). The DNA methylation density of *DCN*, *SFRP4*, *CXCL14*, *STMN2*, *FCN3*, *COMP*, and *LAMA2* was positively, while that of *NPY1R* was negatively correlated with the mRNA expression level of the corresponding genes in HCC (Supplementary Figure 3). Moreover, the methylation level of one CpG site of *DCN*, three CpG sites of *SFRP4*, one CpG site of *MOXD1*, one CpG site of *STMN2*, one CpG site of *COMP*, two CpG sites of *LAMA2*, and two CpG sites of *NPY1R* was significantly associated with OS of HCC patients (Figure 5B).

### Correlations between the expression of DEGs and immune infiltration in HCC

Because immune infiltration plays critical roles in the progression of cancers [20], correlations between the expression of DEGs and the immune infiltration in HCC were investigated by TIMER server. Tumor purity is defined as the proportion of cancer cells in the tumor admixture, which can interfere with the evaluation of immune infiltration. Thus, the correlation analysis of immune infiltration was adjusted with the corresponding tumor purity of samples [21]. As shown in Figure 5, the expression of the 12 DEGs was all negatively correlated to the tumor purity, which suggested the expression of these genes might be mainly from cells in the TME, rather than cancer cells.

**Figure 6:**
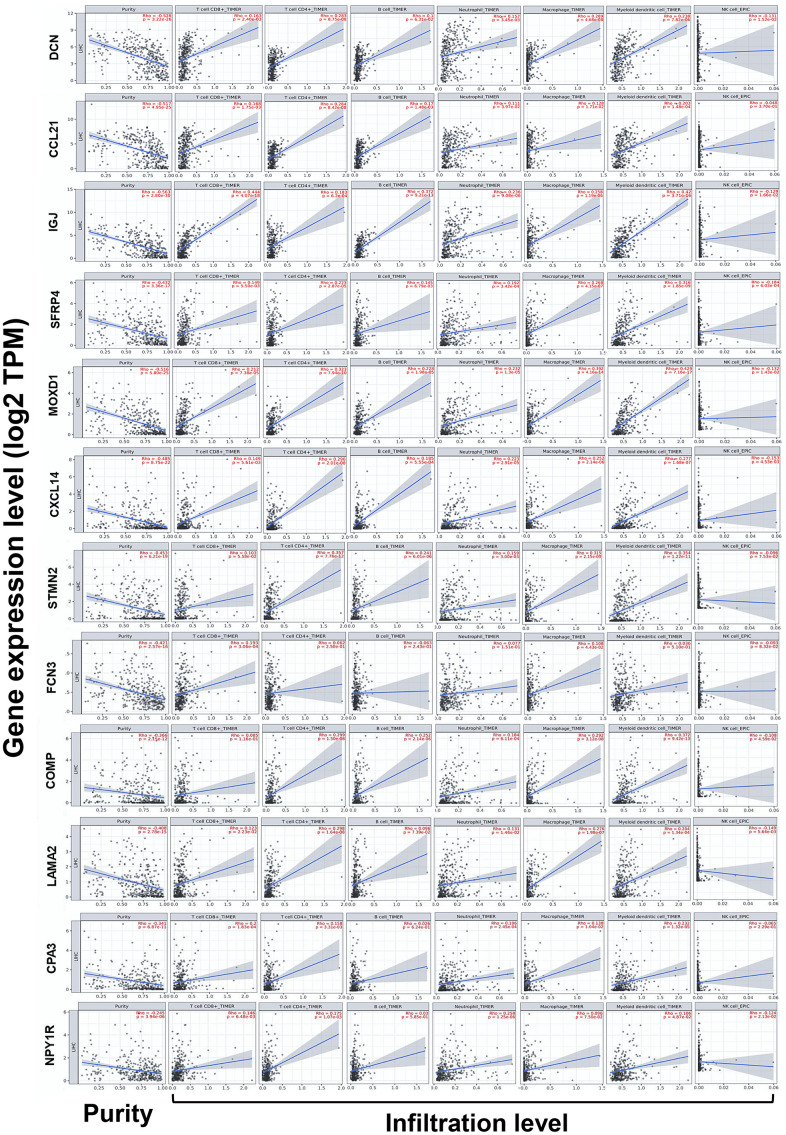
**Correlations between the expression of the DEGs with immune infiltration in HCC (TIMER).** Correlations between the expression of the 12 DEGs with tumor purity, and infiltration level of CD8+ T cells, CD4+ T cells, B cells, neutrophils, macrophages, DCs, and NK cells in HCC. DCs, dendritic cells; NK cells, natural killer cells.

Notably, we found the expression of *DCN*, *CCL21*, *IGJ*, *SFRP4*, *MOXD1*, *CXCL14*, *STMN2*, *COMP*, *LAMA2*, *CPA3*, and *NPY1R* was almost conformably positively correlated with the infiltration level of CD8+ T cells, CD4+ T cells, B cells, neutrophils, macrophages, and DCs, but negatively correlated with that of NK cells (except for *COMP* with CD8+ T cells, *LAMA2* with B cells, *CPA3* with B cells and NK cells, along with *NPY1R* with B cells and macrophages) (*P* < 0.05). In addition, the expression of *FCN3* was positively correlated with the infiltration level of CD8+ T cells and macrophages (Figure 6).

### Intergenic correlations, co-expression network, and functions of the DEGs

**Figure 7:**
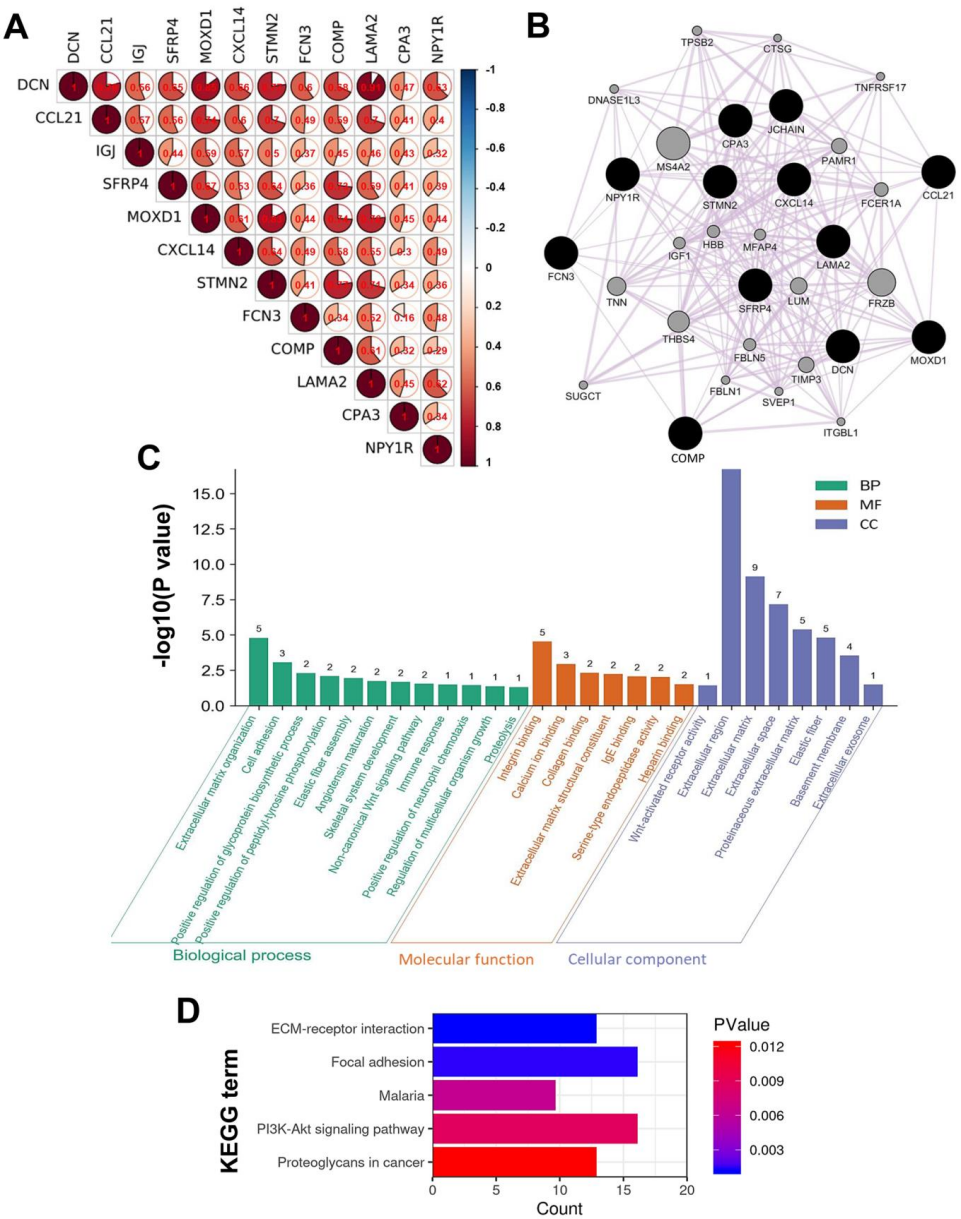
**Intergenic correlations, co-expression network, and the biological functions of the DEGs.** (**A**) Intergenic correlations of the 12 DEGs. (**B**) The co-expression network of the 12 DEGs constructed by GeneMANIA. Edges in the network represent the co-expression relations among genes. The results of (**C**) GO and (**D**) KEGG functional enrichment analyses for all the genes in the co-expression network.

To deeply understand the characters of the DEGs play in HCC, intergenic correlations among the DEGs were analyzed, a co-expression network was constructed, and functional annotation analysis was conducted. The results of intergenic correlation analyses implied the DEGs correlated with each other closely with strong or moderate correlation strength (*P* < 1.00E-05) (Figure 7A). Then, 20 co-expressive genes of the 12 DEGs were identified using GeneMANIA, so the network was composed of 32 genes totally (Figure 7B). The functional enrichment analyses expounded the genes in the co-expression network were components of ECM, and mainly took part in the biological processes of ECM organization, cell adhesion, and glycoprotein synthesis, together with the binding of integrin, calcium ion, collagen, and so on (Figure 7C). Moreover, these genes were involved in signaling pathways of ECM-receptor interaction, focal adhesion, phosphoinositide 3-kinase-protein kinase B (PI3K-Akt), and proteoglycans in cancer (Figure 7D).

## DISCUSSION

The current study identified not many DEGs between PT and PVTT tissues, and a vast majority of them were low-expressed in PVTT compared with paired PT tissues (Figure 1), which was consistent with the previous studies [13, 14]. Among the 12 DEGs, the expression level of *DCN*, *CCL21*, *IGJ*, *CXCL14*, *FCN3*, *LAMA2*, and *NPY1R* was progressively decreased from normal liver, PT, to PVTT (all were significantly except for *LAMA2*), and the up-expression of them linked with favorable survivals of all HCC patients (Figure 3). Also, the upregulation of them (except for *NPY1R*) indicated better outcomes of HCC patients with a history of hepatitis B, though hepatitis B virus infection is an independent risk factor of HCC vascular invasion [15, 22, 23]. Notably, high expression of *NPY1R* implied both favorable OS and PFS of patients in advanced stages or with high-grade tumors (Supplementary Tables 1, 2).

The tumor-suppressive effects of the above seven genes had been illuminated before. DCN is a small leucine-rich proteoglycan acting as a powerful cancer repressor by blocking receptor tyrosine kinases [24], whose expression was decreased in HCC compared with normal liver tissues and followed the staging [25]. Chemokine CCL21 and its unique receptor CCR7 had been described as vital factors determining cancer lymph node metastasis, and they were observed elevated in colorectal liver metastases [26]. IGJ is the joining chain of multimeric IgA and IgM, whose upregulation might augment the anticancer immune responses by Sorafenib treatment and favored survivals of HCC patients [27]. C-x-C motif chemokine CXCL14 was found stably lower regulated in HCC than normal liver tissues [28], whose up-expression could attract DCs, T cells, and NK cells to enhance immunosurveillance, together with inhibiting angiogenesis and aggressiveness in HCC [29, 30]. FCN3 is a member of the ficolin family with lectin activity. It was reported that HCC patients with a higher serologically FCN3 level tended to have longer DFS after radiofrequency ablation treatment [31]. LAMA2 is a kind of ECM protein that was ever found frequently high-allelic mutated in HCC, and a lower expression of *LAMA2* might correlate with a higher chance of recurrence and poorer survivals of HCC patients [32]. NPY1R was ever reported with the capability of restraining HCC cell proliferation via inactivating mitogen-activated protein kinase (MAPK) signaling, and it was usually significantly decreased in HCC [33].

As for the other DEGs, *COMP*, *STMN2*, and *CPA3* were ever reported to play vicious roles in HCC, and *SFRP4* played a benign one, while the character of *MOXD1* in HCC had not been explained yet [34]. COMP is a large pentameric glycoprotein that can promote fibrillogenesis in liver [35], and it might facilitate HCC invasion and metastasis by activating PI3K-Akt signaling [36]. STMN2 is a component of the stathmin family, whose overexpression was critical for maintaining Wnt/β-catenin/TCF mediated hepatic carcinogenesis [37, 38]. CPA3 is a kind of metallo-carboxypeptidase that modulates inflammation, fibrosis, and stem cell niche formation in liver cancer [39], whose overexpression might associate with worse grades, stages, and prognosis of patients [40]. We didn’t find any prognostic significance of *CPA3* in HCC patients, but we found the up-expression of *STMN2* and *COMP* was related to favorable PFS of advanced-stage patients (Supplementary Table 2). The insufficient sample size might cause the paradoxical findings; thus, explorations with a larger sample size are still needed. SFRPs are known as cancer suppressors by block Wnt signaling pathway, whose promoter methylation can reduce the normal expression of SFRP4 and promote HCC [41]. A systematic review demonstrated hypermethylation of *SFRP4* was a risk factor of cancer with an odds ratio (OR) of 11.41 [42]. MOXD1 belongs to the copper-dependent monooxygenase family, whose knockdown would suppress the proliferation of osteosarcoma cells via inducing apoptosis [34]. In the current study, we found methylated CpG sites of *DCN*, *SFRP4*, *MOXD1*, *STMN2*, *COMP*, and *NPY1R* significantly affected OS of HCC patients (Figure 6B).

It could be noticed the alternation of mRNA overexpression occurred frequently in the DEGs (Figure 4B), but most of the DEGs were low-expressed in HCC. Additionally, the DNA methylation of several DEGs was significantly correlated with their expression level (Supplementary Figure 3). Therefore, we suspected that abnormal methylation might induce transcriptional silencing of some DEGs to influence their anticancer or pro-cancer functions, but further validations are required. To sum up, seven DEGs, *DCN*, *CCL21*, *IGJ*, *CXCL14*, *FCN3*, *LAMA2*, and *NPY1R* might play anticancer roles in HCC, whose progressive down-regulation in the liver might promote the initiation of HCC and even venous metastasis. Besides, the DNA methylation of *DCN*, *SFRP4*, *MOXD1*, *STMN2*, *COMP*, and *NPY1R* might also concerned with the progression of HCC.

After that, we observed the expression of most DEGs were significantly correlated with the infiltration density of multiple TIICs (Figure 5). It is acknowledged that CD8+ T cells and NK cells can be motivated by DCs and then exert effective anticancer immune surveillance [43]. Although the role of B cells in HCC remains controversial, the interactions between T cells and B cells might imply better outcomes [44]. CD4+ T cells, tumor-associated macrophages (TAMs), and tumor-associated neutrophils (TANs) act flexibly in HCC, depending on the release of different cytokines and chemokines. TAMs and TANs can polarize into two subtypes respectively, the M1/N1-type ones can amplify anticancer immunity, whereas the M2/N2-type ones act oppositely [45]. Generally speaking, substantial activation of effective TIICs in the TME restrains carcinogenesis and cancer progression [15, 46]. Hence, we could summarize the DEGs might regulate HCC progression partly through modulation of immune infiltration.

The results of alternation occurrence analysis and intergenic correlation analysis implied the DEGs correlated with each other closely (Figure 7A and Supplementary Table 3). And the DEGs’ co-expressive genes were mostly involved in ECM organization, focal adhesion, along with the binding of integrin and collagen (Figure 7), which was consistent with the previous studies [14, 16]. As we know, the TME is formed by cellular components (stroma cells, immune cells, and endothelial cells, etc.), and non-cellular components produced by these cells (ECM, inflammatory cytokines, and growth factors, etc.) [47]. ECM is a complex network performing as a structural scaffold and is mainly consisted of fibrous proteins (eg, collagen) and proteoglycans. The 12 DEGs were components of ECM, which explained the negative correlations between their expression and the tumor purity, since they should be expressed by stroma cells (eg, fibroblasts) in the TME.

For most solid cancers, the metastatic cascade starts with cancer cells breaching the basement membrane and navigating away from the primary site. In some appropriate contexts, the ECM could be an obstacle for cancer metastasis, however, remodeled ECM inclines to be a cancer promoter [48]. Abnormal deposition and stiffness of ECM can promote malignant behaviors of cancer cells, facilitate the colonization of disseminated cancer cells, and mutually interact with immune-suppressive TIICs [49, 50]. Integrins are surface receptors mediating cell-matrix and cell-cell adhesion, which transmit bidirectional signals between cancer cells and the ECM. Integrins are also key components of migration machinery, which determine the colonization sites of metastatic cells and facilitate the survival of these cells [51, 52]. In fact, several pro-survival signals such as PI3K and MAPK pathways should depend on cells that adherent to the ECM via integrins, and the ECM molecules may amplify these signals [53]. In brief, components of the TME interact with each other intricately in cancer progression, our results indicated the DEGs might regulate cancer venous metastasis through alternations of ECM, focal adhesion, and immune infiltration.

## CONCLUSIONS

This study discovered 12 DEGs between PT and PVTT tissues, which might contribute to PVTT development through modulating ECM organization, focal adhesion, and immune infiltration in the TME. The expression of *DCN*, *CCL21*, *IGJ*, *CXCL14*, *FCN3*, *LAMA2*, and *NPY1R* was progressively decreased from normal liver, PT, to PVTT tissues, which might promote the tumorigenesis and venous metastasis of HCC, and whose high-expression might serve as favorable prognostic biomarkers of HCC patients. Additionally, several methylated CpG sites of *DCN*, *SFRP4*, *MOXD1*, *STMN2*, *COMP*, and *NPY1R* might influence outcomes of HCC patients. This study helps to elucidate the molecular mechanisms underlying PVTT formation and provides several genes worthy to further explore.

## MATERIALS AND METHODS

### Data collection

The gene expression data of a total of 36 pairs of human HCC PT and PVTT samples from three datasets were downloaded from the GEO database (https://www.ncbi.nlm.nih.gov/geo/). Datasets GSE69164, GSE77509, and GSE74656 contributed 11, 20, and 5 pairs of samples, respectively. The samples in GSE69164 and GSE77509 were detected by high throughput sequencing using Illumina HiSeq 2000 (GPL11154) and Illumina HiSeq 2500 (GPL16791) respectively; the samples in GSE74656 were processed using GeneChip PrimeView Human Gene Expression Array (GPL16043).

### Data processing and DEGs identification

The mRNA sequencing data were normalized into transcripts per million (TPM) values. Microarray data were normalized into quantile values, and the median value was used as the expression value if several probes matched a single gene. The probe names of the microarray were transformed into gene symbols using the annotation files supplied by the manufacturer. The DEGs between PT and paired PVTT samples in each GEO dataset were analyzed by the “limma” package in R software (Version 4.0.3) with the cutoff criteria of |log_2_(fold change, FC)| > 1 and *P* < 0.05, and the results of which were visualized as volcano plots by the “ggplot2” package [54]. Then, the overlapping DEGs among the three datasets, as identified by Venn diagrams, were regarded as reliable DEGs for our further investigations.

### Analysis of the expression of the DEGs in HCC patients with distinct clinical features

The mRNA expression of the DEGs in HCC and normal liver tissues was analyzed by Gene Expression Profiling Interactive Analysis (GEPIA) web tool (http://gepia.cancer-pku.cn) using data from the Cancer Genome Atlas (TCGA) and the GTEx projects [55]. The significance threshold was set as FC > 2 and *P* < 0.05.

The associations between the expression of the DEGs and clinical features of HCC patients, including genders, ages, stages, and tumor grades were explored using UALCAN (http://ualcan.path.uab.edu), which is an interactive platform for in-depth analysis of cancer omics data from TCGA [56].

### Survival analysis of the DEGs

Kaplan-Meier (KM) Plotter (http://www.kmplot.com/) is an online tool for survival analysis of 54k genes in 21 types of cancers, whose data sources include GEO, TCGA, and European Genome-phenome Archive [57]. Associations between the DEGs’ expression and OS, relapse free survival (RFS), and progression free survival (PFS) of all HCC patients were analyzed by KM Plotter.

Prognostic values of the DEGs on OS and PFS of HCC patients with distinct clinical parameters were also assessed integrating KM Plotter and another prognosis analysis tool, Online consensus Survival for liver hepatocellular carcinoma (OSlihc, http://bioinfo.henu.edu.cn/) [58]. All cases were split into two groups by the median value of a gene’s expression level and univariant analysis was conducted.

### Analysis of genomic alternations of the DEGs

cBioPortal (http://www.cbioportal.org/) is a web resource providing multidimensional cancer genomics data [59, 60]. Genomic alternation profiles of the DEGs including mutations, putative copy-number alterations (CNA), and mRNA expression (z-scores relative to diploid samples with a score threshold of ± 2.0) were analyzed using the data of 360 HCC patients in “TCGA, Firehose Legacy” dataset by cBioPortal. The co-occurrence tendency of pairs of alterations in any two DEGs was analyzed by Fisher’s exact test. Moreover, all available cases were split into altered and unaltered groups, the univariant analysis was conducted to discover the effect of the genetic alternations of the DEGs on survivals of HCC patients.

### DNA methylation-related analysis of the DEGs

DNA Methylation Interactive Visualization Database (DNMIVD, http://www.unimd.org/dnmivd/) [61], SurvivalMeth (http://biobigdata.hrbmu.edu.cn/-survivalmeth/) [62], and MethSurv (https://biit.cs.ut.ee/methsurv/) [63] were all useful web tools providing annotation and survival analysis of DNA methylation in human cancers whose data sources include TCGA and GEO. The global DNA methylation levels of the DEGs in HCC tumor and normal liver tissues were analyzed integrating DNMIVD and SurvivalMeth. Correlations between DNA methylation density and the expression level of the DEGs were analyzed by DNMIVD. Associations between the methylation level of CpG sites of the DEGs and HCC patients’ OS were evaluated by MethSurv. Here, samples were divided into two groups by the median DNA methylation beta value.

### Correlations between the DEGs’ expression and immune infiltration in HCC

Tumor IMmune Estimation Resource (TIMER) (http://timer.cistrome.org) is a web server for the investigation into tumor-immune interactions covering 32 kinds of cancers from TCGA [64]. The correlations between the DEGs expression and infiltration level of diverse tumor-infiltrating immune cells (TIICs), including CD8+ T cells, CD4+ T cells, B cells, neutrophils, macrophages, dendritic cells (DCs), and natural killer (NK) cells in HCC were assessed by TIMER.

### Co-expression analysis of the DEGs and functional enrichment analysis

Intergenic correlations of the DEGs were analyzed using GEPIA platform. A co-expression network of the DEGs was constructed using GeneMANIA (http://genemania.org) [65]. Gene Ontology (GO) and Kyoto Encyclopedia of Genes and Genomes (KEGG) pathway enrichment analysis was performed for the component genes in the co-expression network, using Database for Annotation, Visualization, and Integrated Discovery (DAVID) server (https://david.ncifcrf.gov/home.jsp) [66].

### Statistical analysis

The student’s t-test was applied to compare differences in mRNA expression or DNA methylation of genes between two kinds of tissues. Kaplan-Meier curves and log-rank test were performed to explore the associations between expression or DNA methylation of genes and patients’ survivals. Spearman’s method was applied to evaluate the intergenic correlations, correlations between gene expression and DNA methylation, or correlations between gene expression and immune infiltration. Correlation strength was measured by correlation coefficient values: 0.00 - 0.19 was “very weak”, 0.20 - 0.39 was “weak”, 0.40 - 0.59 was “moderate”, 0.60 - 0.79 was “strong”, and 0.80 -1.0 was “very strong” [67, 68]. All tests were two-tailed paired and *P* values < 0.05 were considered statistically significant.

### Data availability statement

All the data that support the findings of this study are publicly available in: https://www.oncomine.org/resource/login.html, http://gepia.cancer-pku.cn, http://ualcan.path.uab.edu, http://www.cbioportal.org/, http://www.kmplot.com/, http://bioinformatica.mty.itesm.mx:8080/Biomatec/SurvivaX.jsp, http://timer.cistrome.org, http://www.unimd.org/dnmivd/, http://biobigdata.hrbmu.edu.cn/survivalmeth/, https://biit.cs.ut.ee/methsurv/, http://genemania.org, https://david.ncifcrf.gov/home.jsp, together with the Supplementary Materials.

## Supplementary Material

Supplementary Figures

Supplementary Table 1

Supplementary Table 2

Supplementary Table 3

Supplementary Table 4
