# Supplementary material for: Identification of prognostic biomarkers associated with the occurrence of portal vein tumor thrombus in hepatocellular carcinoma
Source: Aging (Albany NY). 2021 Apr 20;13(8):11786–807. doi: 10.18632/aging.202876 (PMC8109071; doi:10.18632/aging.202876)
Supplement: Supplementary Figures [file aging-13-202876-s001.pdf]

SUPPLEMENTARY FIGURES

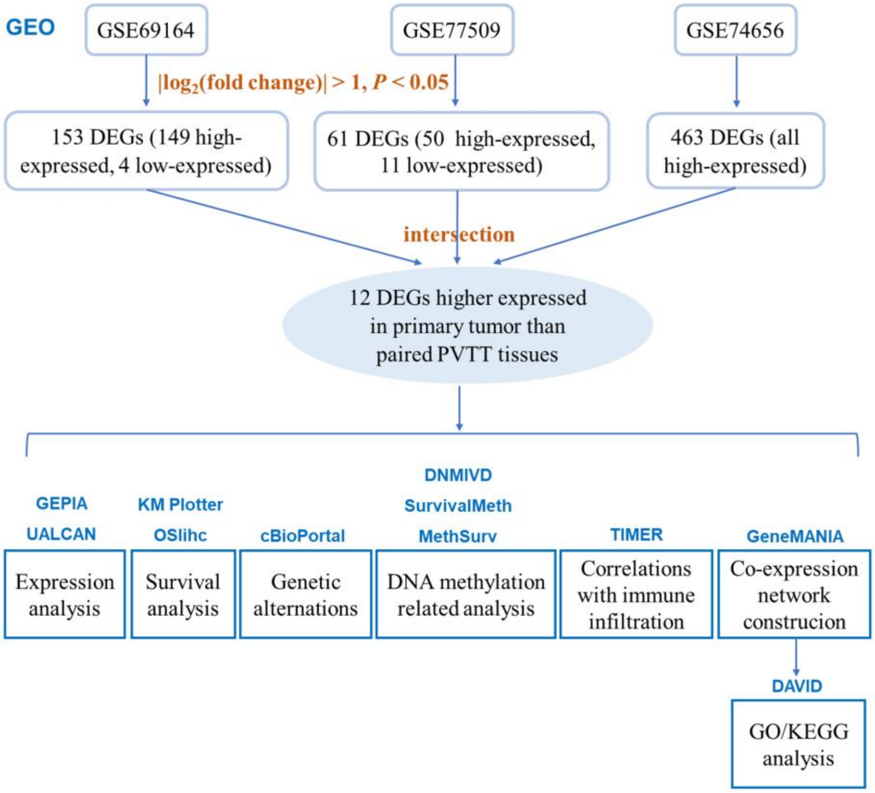

Supplementary Figure 1. The workflow of this study.

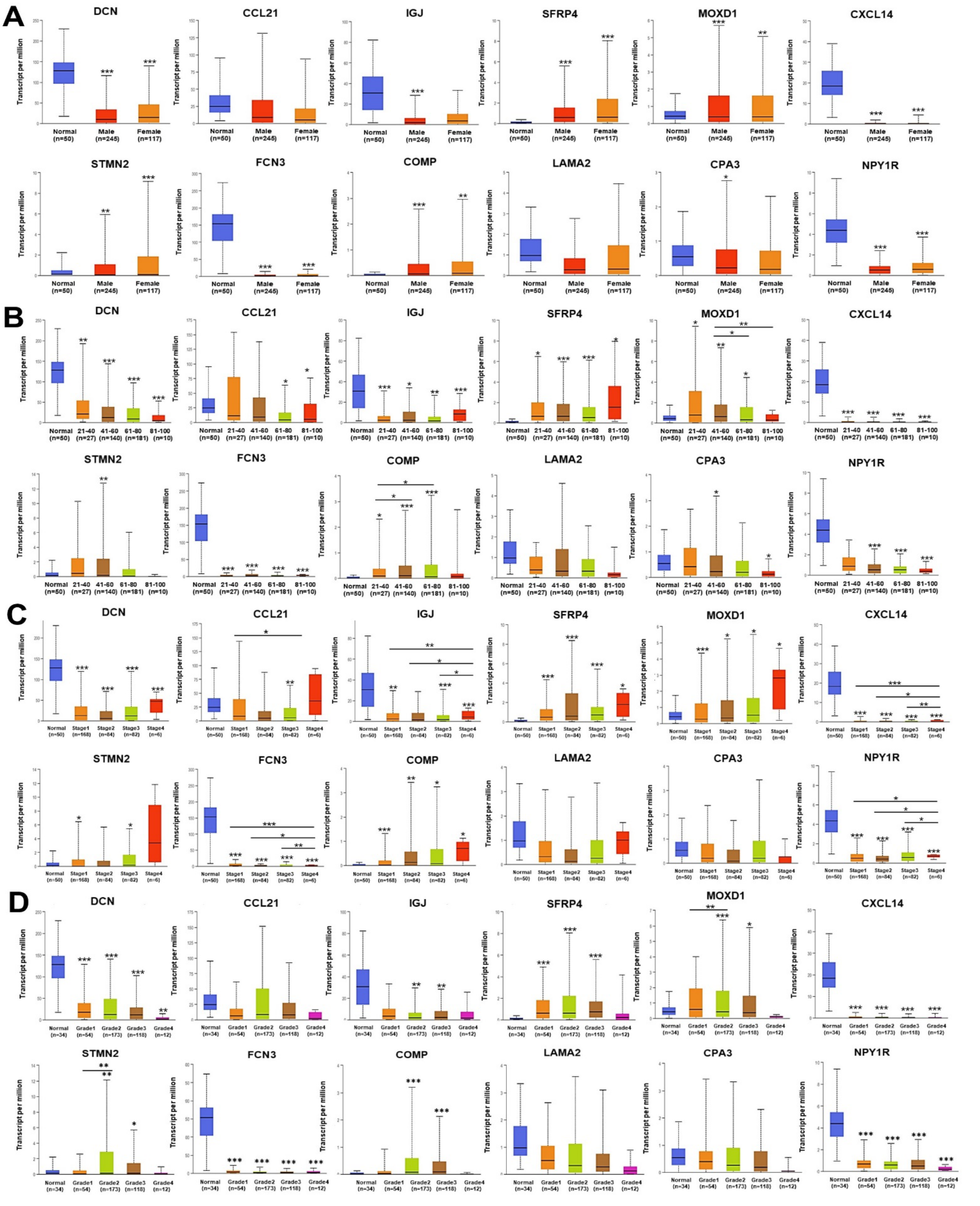

Supplementary Figure 2. Expression of the 12 DEGs in HCC patients with distinct clinical parameters (UALCAN). Expression of the 12 DEGs in HCC patients classified by (**A**) genders, (**B**) ages, (**C**) stages, and (**D**) grades. (*P < 0.05, **P < 0.01, ***P < 0.001).

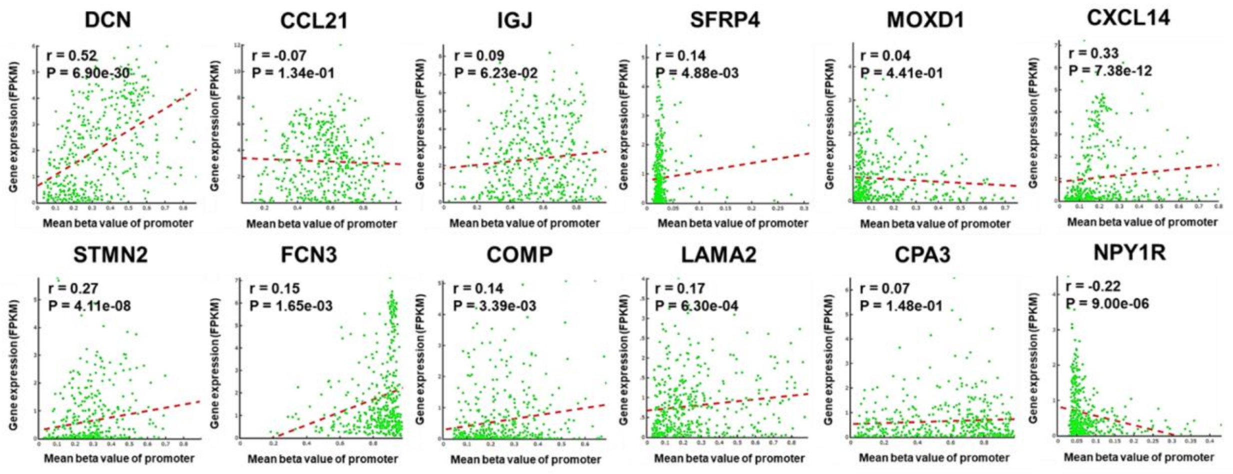

Supplementary Figure 3. The correlations between DNA methylation density and the mRNA expression level of the DEGs in HCC (DNMIVD).
