# Supplementary material for: Identification of prognostic biomarkers associated with the occurrence of portal vein tumor thrombus in hepatocellular carcinoma
Source: Aging (Albany NY). 2021 Apr 20;13(8):11786–807. doi: 10.18632/aging.202876 (PMC8109071; doi:10.18632/aging.202876)
Supplement: Supplementary Table 1 [file aging-13-202876-s002.docx]

Supplementary Table 1. Associations between the DEGs’ expression with OS of HCC patients with diverse clinical characteristics.

| Gene symbol | | *DCN* | | *CCL21* | | *IGJ* | | *SFRP4* | | *MOXD1* | | *CXCL14* | |
| --- | --- | --- | --- | --- | --- | --- | --- | --- | --- | --- | --- | --- | --- |
| Clinical parameters | *N* | HR (95%CI) | *P* | HR (95%CI) | *P* | HR (95%CI) | *P* | HR (95%CI) | *P* | HR (95%CI) | *P* | HR (95%CI) | *P* |
| **Gender** |  |  |  |  |  |  |  |  |  |  |  |  |  |
| Male | 146 | 0.73(0.47-1.13) | 0.156 | 0.78(0.5-1.21) | 0.262 | 0.68(0.43-1.06) | 0.083 | 1.05 (0.68-1.64) | 0.818 | 1.16(0.75-181) | 0.500 | 0.87(0.56-1.35) | 0.528 |
| Female | 118 | 0.66(0.38-1.14) | 0.130 | 0.72(0.41-1.25) | 0.240 | 1.01(0.58-1.76) | 0.960 | 1.45(0.83-2.53) | 0.190 | 0.95(0.55-1.66) | 0.870 | 1.10(0.63-1.91) | 0.740 |
| **Family history** | |  |  |  |  |  |  |  |  |  |  |  |  |
| Yes | 109 | 0.72(0.41-1.27) | 0.254 | 0.62(0.35-1.10) | 0.101 | 0.63(0.37-1.08) | 0.091 | 0.84(0.48-1.49) | 0.560 | 1.03(0.58-1.81) | 0.923 | 0.79(0.45-1.40) | 0.415 |
| No | 203 | 0.65(0.40-1.05) | 0.076 | 0.79(0.49-1.28) | 0.343 | 0.61(0.41-0.90) | **0.014** | 1.14(0.71-1.84) | 0.593 | 0.65(0.40-1.05) | 0.489 | 1.08(0.67-1.73) | 0.753 |
| **Risk factors** | |  |  |  |  |  |  |  |  |  |  |  |  |
| Alcohol consumption | 76 | 0.51(0.25-1.07) | 0.069 | 0.56(0.27-1.16) | 0.116 | 0.69(0.34-1.42) | 0.312 | 0.57(0.27-1.19) | 0.131 | 0.64(0.31-1.35) | 0.243 | 0.92(0.44-1.91) | 0.814 |
| Hepatitis B | 75 | 0.31(0.11-0.89) | **0.028** | 0.38(0.14-1.04) | 0.059 | 0.31(0.11-0.89) | **0.030** | 1.42(0.54-3.73) | 0.480 | 0.79(0.31-2.06) | 0.636 | 0.21(0.07-0.66) | **0.007** |
| Hepatitis C | 32 | 0.73(0.22-2.43) | 0.609 | 0.91(0.26-3.15) | 0.881 | 1.71(0.48-6.09) | 0.409 | 0.54(0.16-1.83) | 0.322 | 0.75(0.23-2.50) | 0.642 | 1.97(0.55-7.12) | 0.298 |
| None | 91 | 0.67(0.37-1.21) | 0.185 | 0.92(0.51-1.65) | 0.768 | 0.72(0.40-1.3) | 0.273 | 0.86(0.48-1.54) | 0.603 | 1.28 (0.7 -2.33) | 0.417 | 1.26(0.7-2.28) | 0.434 |
| **Stage** |  |  |  |  |  |  |  |  |  |  |  |  |  |
| Ⅰ | 170 | 0.97(0.53-1.78) | 0.917 | 0.96(0.52-1.76) | 0.901 | 0.78(0.43-1.44) | 0.430 | 1.42(0.76-2.63) | 0.268 | 1.05(0.57-1.93) | 0.869 | 0.72(0.39-1.33) | 0.288 |
| Ⅱ | 83 | 0.71(0.32-1.59) | 0.409 | 1.00(0.46-2.18) | 0.995 | 0.67(0.31-1.46) | 0.314 | 0.97(0.45-2.11) | 0.941 | 1.44(0.65-3.19) | 0.363 | 1.17(0.54-2.52) | 0.695 |
| Ⅲ+Ⅳ | 87 | 0.62(0.35-1.09) | 0.095 | 0.84(0.47-1.49) | 0.540 | 0.85(0.48-1.52) | 0.584 | 0.87(0.49-1.53) | 0.620 | 0.66(0.37-1.18) | 0.162 | 0.90(0.5-1.59) | 0.706 |
| **Grade** |  |  |  |  |  |  |  |  |  |  |  |  |  |
| 1 | 55 | 0.98(0.38-2.56) | 0.972 | 0.82(0.31-2.17) | 0.686 | 0.78(0.31-1.98) | 0.600 | 1.25(0.49-3.2) | 0.644 | 0.94(0.37-2.38) | 0.888 | 1.08(0.42-2.76) | 0.881 |
| 2 | 174 | 0.66(0.39-1.11) | 0.116 | 0.78(0.47-1.29) | 0.332 | 0.78(0.47-1.31) | 0.346 | 0.92(0.55-1.53) | 0.735 | 1.23(0.73-2.06) | 0.434 | 1.21(0.73-2.02) | 0.463 |
| 3 | 118 | 0.91(0.5-1.65) | 0.750 | 0.80(0.44-1.45) | 0.460 | 0.66(0.36-1.20) | 0.168 | 1.44(0.79-2.65) | 0.234 | 1.06(0.58-1.94) | 0.841 | 0.62(0.34-1.15) | 0.126 |
| Gene symbol | | *STMN2* | | *FCN3* | | *COMP* | | *CPA3* | | *LAMA2* | | *NPY1R* | |
| Clinical parameters | *N* | HR (95%CI) | *P* | HR (95%CI) | *P* | HR (95%CI) | *P* | HR (95%CI) | *P* | HR (95%CI) | *P* | HR (95%CI) | *P* |
| **Gender** |  |  |  |  |  |  |  |  |  |  |  |  |  |
| Male | 146 | 1.23(0.79-1.92) | 0.351 | 0.60(0.39-0.95) | **0.026** | 1.13(0.73-1.76) | 0.584 | 0.87 (0.56-1.37) | 0.550 | 0.81 (0.52-1.27) | 0.360 | 0.53(0.34-0.83) | **0.005** |
| Female | 118 | 1.02(0.59-1.77) | 0.952 | 0.83(0.47-1.45) | 0.508 | 0.92(0.53-1.60) | 0.763 | 0.87(0.48-1.56) | 0.635 | 0.87 (0.48-1.56) | 0.630 | 0.84(0.48-1.45) | 0.520 |
| **Family history** | |  |  |  |  |  |  |  |  |  |  |  |  |
| Yes | 109 | 0.99(0.56-1.75) | 0.979 | 0.73(0.41-1.30) | 0.287 | 0.86(0.49-1.52) | 0.601 | 0.64(0.36-1.15) | 0.136 | 0.68(0.39-1.21) | 0.188 | 0.38(0.21-0.70) | **0.002** |
| No | 203 | 1.24(0.77-1.99) | 0.374 | 0.61(0.38-0.99) | **0.044** | 1.21(0.75-1.95) | 0.427 | 0.9(0.56-1.48) | 0.700 | 0.76(0.47-1.22) | 0.260 | 0.82(0.51-1.31) | 0.406 |
| **Risk factors** | |  |  |  |  |  |  |  |  |  |  |  |  |
| Alcohol consumption | 76 | 0.7(0.34-1.45) | 0.339 | 0.21(0.08-0.52) | **<0.001** | 0.58(0.28-1.20) | 0.138 | 0.58(0.28-1.22) | 0.150 | 0.5(0.24-1.04) | 0.058 | 0.47 (0.23-0.98) | **0.040** |
| Hepatitis B | 75 | 0.55(0.21-1.45) | 0.226 | 0.83(0.32-2.16) | 0.705 | 0.81(0.31-2.11) | 0.668 | 0.54(0.20-1.43) | 0.213 | 0.31(0.12-1.09) | **0.029** | 0.54(0.20-1.44) | 0.218 |
| Hepatitis C | 32 | 1.22(0.37-4.07) | 0.745 | 1.61(0.46-5.59) | 0.454 | 0.92(0.28-3.06) | 0.890 | 0.88(0.27-2.91) | 0.839 | 0.73(0.22-2.43) | 0.609 | 1.01(0.29-3.51) | 0.990 |
| None | 91 | 1.59 (0.87-2.9) | 0.127 | 0.60(0.33-1.09) | 0.092 | 1.55(0.86-2.79) | 0.145 | 0.94(0.51-1.72) | 0.840 | 0.98(0.54-1.76) | 0.945 | 0.48(0.26-0.87) | **0.014** |
| **Stage** |  |  |  |  |  |  |  |  |  |  |  |  |  |
| Ⅰ | 170 | 1.06(0.58-1.94) | 0.853 | 0.68(0.37-1.25) | 0.212 | 0.74(0.40-1.37) | 0.336 | 0.84(0.45-1.57) | 0.586 | 0.96(0.53-1.77) | 0.909 | 0.82(0.44-1.5) | 0.511 |
| Ⅱ | 83 | 1.15(0.53-2.49) | 0.724 | 0.49(0.22-1.09) | 0.075 | 0.94(0.43-2.04) | 0.881 | 1.27(0.58-2.80) | 0.554 | 0.47(0.2-1.08) | 0.069 | 0.47(0.2-1.08) | 0.067 |
| Ⅲ+Ⅳ | 87 | 0.78(0.44-1.39) | 0.402 | 0.81(0.46-1.43) | 0.458 | 0.9(0.51-1.59) | 0.709 | 0.84(0.47-1.51) | 0.565 | 0.65(0.37-1.15) | 0.139 | 0.39(0.22-0.71) | **0.002** |
| **Grade** |  |  |  |  |  |  |  |  |  |  |  |  |  |
| 1 | 55 | 2.02(0.74-5.46) | 0.159 | 0.96(0.37-2.55) | 0.942 | 1.67(0.63-4.43) | 0.298 | 0.47(0.18-1.24) | 0.118 | 1.93(0.71-5.28) | 0.190 | 0.61(0.24-1.56) | 0.296 |
| 2 | 174 | 0.96(0.57-1.6) | 0.878 | 0.58(0.34-0.97) | **0.037** | 1.02(0.61-1.70) | 0.933 | 1.35(0.81-2.24) | 0.252 | 0.74(0.43-1.24) | 0.250 | 0.61(0.37-102) | 0.059 |
| 3 | 118 | 1.14(0.62-2.07) | 0.671 | 0.77(0.42-1.41) | 0.393 | 0.98(0.54-1.78) | 0.938 | 0.95 (0.52-1.73) | 0.863 | 0.79(0.43-1.44) | 0.442 | 0.51(0.27-0.95) | **0.032** |

*Note*: The results with statistical significance were in bold. The results for the patients with a history of hepatitis B/C, and the patients with/without a family history of cancer were analyzed by OSlihc; the others were analyzed by KM Plotter. Abbreviations: OS, overall survival; HR, hazard ratio; CI, confidence interval.
