# Supplementary material for: Identification of prognostic biomarkers associated with the occurrence of portal vein tumor thrombus in hepatocellular carcinoma
Source: Aging (Albany NY). 2021 Apr 20;13(8):11786–807. doi: 10.18632/aging.202876 (PMC8109071; doi:10.18632/aging.202876)
Supplement: Supplementary Table 2 [file aging-13-202876-s003.docx]

Supplementary Table 2. Associations between the DEGs’ expression with PFS of HCC patients with diverse clinical characteristics.

| Gene symbol | | *DCN* | | *CCL21* | | *IGJ* | | *SFRP4* | | *MOXD1* | | *CXCL14* | |
| --- | --- | --- | --- | --- | --- | --- | --- | --- | --- | --- | --- | --- | --- |
| Clinical parameters | *N* | HR (95%CI) | *P* | HR (95%CI) | *P* | HR (95%CI) | *P* | HR (95%CI) | *P* | HR (95%CI) | *P* | HR (95%CI) | *P* |
| **Gender** |  |  |  |  |  |  |  |  |  |  |  |  |  |
| Male | 250 | 0.62(043-0.89) | **0.009** | 0.63(0.44-0.91) | **0.012** | 0.66(0.46-0.95) | **0.023** | 0.82(0.57-1.17) | 0.277 | 0.78(0.54-1.12) | 0.179 | 0.89(0.62-1.27) | 0.524 |
| Female | 121 | 1.08(0.65-1.79) | 0.774 | 0.66(0.4-1.1) | 0.110 | 0.67(0.40-1.11) | 0.116 | 1.48(0.89-2.48) | 0.131 | 1.19(0.71-1.98) | 0.506 | 0.69(0.42-1.16) | 0.162 |
| **Family history** | |  |  |  |  |  |  |  |  |  |  |  |  |
| Yes | 109 | 0.82(0.48-1.39) | 0.456 | 0.80(0.47-1.38) | 0.424 | 0.56(0.33-0.96) | **0.035** | 0.96(0.56-1.64) | 0.880 | 0.85(0.50-11.46) | 0.554 | 0.81(0.47-1.39) | 0.442 |
| No | 203 | 0.61(0.41-0.91) | **0.014** | 1.77(1.08-2.88) | **0.023** | 0.63(0.42-0.93) | **0.021** | 0.96(0.65-1.41) | 0.830 | 0.97(0.66-1.43) | 0.875 | 1.15(0.78-1.69) | 0.495 |
| **Risk factors** | |  |  |  |  |  |  |  |  |  |  |  |  |
| Alcohol consumption | 78 | 0.59(0.32-1.07) | 0.078 | 0.92(0.51-1.66) | 0.788 | 0.63(0.34-1.15) | 0.131 | 0.91(0.50-1.65) | 0.751 | 0.65(0.36-1.19) | 0.161 | 0.74(0.40-1.36) | 0.332 |
| Hepatitis B | 75 | 0.32(0.16-0.67) | **0.002** | 0.47(0.24-0.90) | **0.031** | 0.43(0.22-0.87) | **0.018** | 0.80(0.40-1.59) | 0.532 | 0.51(0.26-1.02) | 0.058 | 0.44(0.22-0.87) | **0.019** |
| Hepatitis C | 32 | 2.99(0.60-14.81) | 0.180 | 1.09(0.46-2.60) | 0.842 | 1.08(0.45-2.58) | 0.859 | 1.05(0.44-2.53) | 0.905 | 0.96(0.40-2.29) | 0.927 | 1.52(0.62-3.68) | 0.358 |
| None | 91 | 1.21(0.63-2.31) | 0.574 | 0.66(0.34-1.28) | 0.213 | 0.60(0.31-1.15) | 0.123 | 1.50(0.78-2.90) | 0.221 | 1.97(0.99-3.90) | **0.048** | 1.11(0.58-2.13) | 0.742 |
| **Stage** |  |  |  |  |  |  |  |  |  |  |  |  |  |
| Ⅰ | 171 | 0.83(0.51-1.36) | 0.464 | 0.86(0.52-1.41) | 0.547 | 0.90(0.55-1.47) | 0.667 | 1.33(0.81-2.19) | 0.257 | 1.04(0.63-1.70) | 0.886 | 0.79(0.48-1.30) | 0.359 |
| Ⅱ | 85 | 0.72(0.40-1.30) | 0.273 | 0.92(0.51-1.67) | 0.793 | 0.55(0.30-1.00) | **0.048** | 0.76(0.42-1.37) | 0.351 | 0.89(0.49-1.63) | 0.710 | 0.69(0.38-1.28) | 0.241 |
| Ⅲ+Ⅳ | 90 | 0.64(0.38-1.08) | 0.092 | 0.79 (0.47-1.33) | 0.372 | 0.47(0.27-0.81) | **0.005** | 0.9 (0.53-1.52) | 0.687 | 0.60(0.35-1.03) | 0.060 | 0.77(0.46-1.31) | 0.340 |
| **Grade** |  |  |  |  |  |  |  |  |  |  |  |  |  |
| 1 | 55 | 0.50(0.22-1.10) | 0.080 | 0.74(0.34-1.61) | 0.439 | 0.38(0.16-0.86) | **0.017** | 0.65(0.30-1.44) | 0.286 | 0.76(0.35-1.67) | 0.493 | 0.76(0.35-1.66) | 0.488 |
| 2 | 177 | 0.71(0.46-1.1) | 0.120 | 0.65(0.42-1.01) | 0.051 | 0.70(0.45-1.08) | 0.106 | 0.81(0.53-1.26) | 0.355 | 0.99(0.64-1.53) | 0.970 | 0.89(0.57-1.38) | 0.600 |
| 3 | 121 | 0.71(0.43-1.16) | 0.170 | 0.7(0.42-1.14) | 0.151 | 0.68(0.41-1.12) | 0.130 | 1.28(0.78-2.11) | 0.330 | 0.85(0.52-1.40) | 0.520 | 0.64(0.39-1.06) | 0.081 |
| Gene symbol | | *STMN2* | | *FCN3* | | *COMP* | | *CPA3* | | *LAMA2* | | *NPY1R* | |
| Clinical parameters | *N* | HR (95%CI) | *P* | HR (95%CI) | *P* | HR (95%CI) | *P* | HR (95%CI) | *P* | HR (95%CI) | *P* | HR (95%CI) | *P* |
| **Gender** |  |  |  |  |  |  |  |  |  |  |  |  |  |
| Male | 250 | 0.81(0.56-1.16) | 0.251 | 0.67(0.47-0.97) | **0.031** | 0.78(0.54-1.12) | 0.172 | 0.84(0.59-1.21) | 0.351 | 0.63(0.44-0.91) | **0.012** | 0.76(0.53-1.08) | 0.127 |
| Female | 121 | 1.27 (0.76-2.12) | 0.365 | 0.68(0.4-1.13) | 0.132 | 1.02(0.61-1.70) | 0.940 | 0.89(0.54-1.49) | 0.670 | 1.09 (0.66-1.82) | 0.734 | 0.88(0.53-1.46) | 0.612 |
| **Family history** | |  |  |  |  |  |  |  |  |  |  |  |  |
| Yes | 109 | 0.93(0.54-1.59) | 0.784 | 0.66(0.38-1.13) | 0.127 | 0.81(0.47-1.39) | 0.441 | 1.04(0.61-1.79) | 0.882 | 1.20(0.70-2.07) | 0.514 | 0.84(0.49-1.44) | 0.525 |
| No | 203 | 1.02(0.69-1.50) | 0.935 | 0.66(0.44-0.98) | **0.037** | 0.89(0.60-1.31) | 0.555 | 0.86(0.58-1.26) | 0.433 | 0.63(0.425-0.93) | **0.020** | 0.73(0.49-1.08) | 0.117 |
| **Risk factors** | |  |  |  |  |  |  |  |  |  |  |  |  |
| Alcohol consumption | 78 | 0.60(0.33-1.11) | 0.098 | 0.62(0.33-1.16) | 0.129 | 0.87(0.48-1.57) | 0.635 | 1.04(0.57-1.88) | 0.899 | 0.53(0.29-0.96) | **0.032** | 0.76(0.42-1.38) | 0.368 |
| Hepatitis B | 75 | 0.55(0.28-1.20) | 0.092 | 0.49(0.24-0.98) | **0.044** | 0.58(0.29-1.16) | 0.126 | 0.58(0.29-1.15) | 0.120 | 0.36(0.18-0.88) | **0.004** | 0.75(0.38-1.48) | 0.411 |
| Hepatitis C | 32 | 1.01(0.42-2.42) | 0.988 | 0.85(0.36-2.02 | 0.717 | 1.22(0.51-2.90) | 0.653 | 1.23(0.52-2.92) | 0.635 | 1.20(0.50-2.86) | 0.683 | 0.79(0.33-1.89) | 0.598 |
| None | 91 | 2.13(1.07-4.24) | **0.028** | 0.69(036-1.32) | 0.261 | 1.59(0.82-3.08) | 0.165 | 0.98 (0.5-1.87) | 0.948 | 1.61 (0.83 − 3.13) | 0.159 | 0.72(0.38-1.38) | 0.317 |
| **Stage** |  |  |  |  |  |  |  |  |  |  |  |  |  |
| Ⅰ | 171 | 0.99(0.60-1.63) | 0.973 | 0.9(0.55-1.48) | 0.690 | 0.79(0.48-1.30) | 0.359 | 1.34(0.81-2.21) | 0.255 | 0.87(0.53-1.43) | 0.590 | 0.96(0.59-1.58) | 0.887 |
| Ⅱ | 85 | 1.12(0.62-2.01) | 0.708 | 0.67(0.37-1.21) | 0.184 | 0.81(0.45-1.47) | 0.487 | 0.65(0.35-1.19) | 0.156 | 0.81(0.45-1.46) | 0.480 | 0.94(0.52-1.69) | 0.835 |
| Ⅲ+Ⅳ | 90 | 0.51(0.30-0.88) | **0.013** | 0.74(0.44-1.25) | 0.254 | 0.58(0.34-0.99) | **0.044** | 0.84 (0.5-1.43) | 0.522 | 0.71(0.42-1.2) | 0.194 | 0.52(0.30-0.89) | **0.014** |
| **Grade** |  |  |  |  |  |  |  |  |  |  |  |  |  |
| 1 | 55 | 0.64(0.29-1.41) | 0.260 | 0.43(0.19-0.97) | **0.037** | 0.6(0.27-1.34) | 0.209 | 0.6(0.27-1.32) | 0.200 | 0.92(0.43-2) | 0.840 | 0.97(0.44-2.12) | 0.940 |
| 2 | 177 | 1.00(0.65-1.54) | 1.000 | 0.64(0.42-1) | **0.048** | 0.92(0.6-1.43) | 0.720 | 0.96(0.62-1.48) | 0.840 | 0.72(0.46-1.11) | 0.130 | 0.68(0.44-1.06) | 0.084 |
| 3 | 121 | 0.84(0.51-1.39) | 0.500 | 0.74(0.45-1.21) | 0.230 | 1.02(0.62-1.68) | 0.930 | 0.76(0.46-1.25) | 0.280 | 0.74(045-1.22) | 0.240 | 0.74(0.45-1.22) | 0.240 |

*Note*: The results with statistical significance were in bold. The results for the patients with a history of hepatitis B/C, and the patients with/without a family history of cancer were analyzed by OSlihc; the others were analyzed by KM Plotter. Abbreviations: PFS, progression free survival; HR, hazard ratio; CI, confidence interval.
