# Supplementary material for: Identification of prognostic biomarkers associated with the occurrence of portal vein tumor thrombus in hepatocellular carcinoma
Source: Aging (Albany NY). 2021 Apr 20;13(8):11786–807. doi: 10.18632/aging.202876 (PMC8109071; doi:10.18632/aging.202876)
Supplement: Supplementary Table 3 [file aging-13-202876-s004.pdf]

**Supplementary Table 3. Co-occurrence tendency of pairs of alterations of the DEGs (cBioPortal).**

| Gene1 | Gene2 | Log2 odds ratio | P value | Q value | Tendency |
|---|---|---|---|---|---|
| DCN | FCN3 | >3 | <0.001 | <0.001 | co-occurrence |
| MOXD1 | LAMA2 | >3 | <0.001 | <0.001 | co-occurrence |
| DCN | MOXD1 | >3 | <0.001 | <0.001 | co-occurrence |
| DCN | CXCL14 | >3 | <0.001 | 0.001 | co-occurrence |
| FCN3 | LAMA2 | >3 | <0.001 | 0.003 | co-occurrence |
| SFRP4 | COMP | >3 | <0.001 | 0.005 | co-occurrence |
| DCN | LAMA2 | 2.676 | <0.001 | 0.009 | co-occurrence |
| SFRP4 | STMN2 | >3 | 0.001 | 0.009 | co-occurrence |
| IGJ | CXCL14 | >3 | 0.003 | 0.02 | co-occurrence |
| IGJ | CPA3 | >3 | 0.003 | 0.023 | co-occurrence |
| STMN2 | NPY1R | 2.644 | 0.004 | 0.024 | co-occurrence |
| MOXD1 | NPY1R | >3 | 0.008 | 0.046 | co-occurrence |

*Note*: Odds ratio = (odds of alteration in Gene2 given alteration in Gene1)/(odds of alteration in Gene2 given lack of alteration in Gene1). A positive value of "Log2 odds ratio" suggests alternations in a pair of genes co-occur in the same samples, while a negative value suggests alternations in a pair of genes are mutually exclusive and tend to occur in different samples.
