# Supplementary material for: Identification of prognostic biomarkers associated with the occurrence of portal vein tumor thrombus in hepatocellular carcinoma
Source: Aging (Albany NY). 2021 Apr 20;13(8):11786–807. doi: 10.18632/aging.202876 (PMC8109071; doi:10.18632/aging.202876)
Supplement: Supplementary Table 4 [file aging-13-202876-s005.docx]

Supplementary Table 4. Significant differential methylation-related functional elements of the DEGs.

| Gene Symbol | CpG (probe ID) | Group | Relation to  island | Average of  tumor samples | Average of  normal samples | Delta value | Fold change | *P* value |
| --- | --- | --- | --- | --- | --- | --- | --- | --- |
| *DCN* | cg00608965 | Body;1stExon | OpenSea | 0.403 | 0.639 | -0.237 | 0.630 | 3.96E-46 |
| *DCN* | cg02442619 | Body;1stExon | OpenSea | 0.228 | 0.359 | -0.131 | 0.635 | 1.07E-14 |
| *DCN* | cg04018214 | Body;1stExon | OpenSea | 0.447 | 0.696 | -0.249 | 0.642 | 1.30E-38 |
| *DCN* | cg04088433 | 1stExon;5'UTR | OpenSea | 0.403 | 0.636 | -0.233 | 0.633 | 1.09E-35 |
| *DCN* | cg05179204 | Body;3'UTR | OpenSea | 0.539 | 0.870 | -0.330 | 0.620 | 1.87E-73 |
| *DCN* | cg13237265 | Body;3'UTR | OpenSea | 0.564 | 0.882 | -0.317 | 0.640 | 1.79E-72 |
| *DCN* | cg13562816 | Body | OpenSea | 0.354 | 0.636 | -0.282 | 0.556 | 7.67E-42 |
| *DCN* | cg15395461 | Body;3'UTR | OpenSea | 0.711 | 0.909 | -0.198 | 0.782 | 1.11E-45 |
| *DCN* | cg26985711 | Body;1stExon | OpenSea | 0.192 | 0.336 | -0.144 | 0.571 | 1.72E-30 |
| *CCL21* | cg27443224 | 1stExon;5'UTR | OpenSea | 0.534 | 0.602 | -0.069 | 0.886 | 7.23E-08 |
| *IGJ* | cg03968222 | 3'UTR | OpenSea | 0.700 | 0.828 | -0.128 | 0.845 | 3.46E-23 |
| *IGJ* | cg17075352 | TSS1500 | OpenSea | 0.515 | 0.625 | -0.110 | 0.824 | 2.13E-09 |
| *SFRP4* | cg01689311 | TSS1500 | S_Shore | 0.058 | 0.042 | 0.016 | 1.374 | 8.88E-14 |
| *SFRP4* | cg04651042 | TSS1500 | S_Shore | 0.049 | 0.058 | -0.010 | 0.836 | 6.40E-07 |
| *SFRP4* | cg05682561 | TSS1500 | S_Shore | 0.049 | 0.046 | 0.003 | 1.065 | 0.046656 |
| *SFRP4* | cg06161814 | TSS1500 | S_Shore | 0.060 | 0.074 | -0.015 | 0.800 | 1.90E-05 |
| *SFRP4* | cg09594069 | TSS1500 | S_Shore | 0.069 | 0.084 | -0.016 | 0.815 | 9.27E-07 |
| *SFRP4* | cg11878069 | 1stExon;5'UTR | Island | 0.022 | 0.018 | 0.004 | 1.248 | 0.032774 |
| *SFRP4* | cg13400306 | 3'UTR | OpenSea | 0.808 | 0.886 | -0.078 | 0.912 | 1.32E-12 |
| *SFRP4* | cg14846368 | Body | Island | 0.421 | 0.331 | 0.091 | 1.275 | 1.12E-05 |
| *SFRP4* | cg18723937 | 1stExon | Island | 0.413 | 0.204 | 0.209 | 2.027 | 2.16E-19 |
| *SFRP4* | cg19166347 | Body | N_Shore | 0.326 | 0.240 | 0.086 | 1.356 | 2.11E-11 |
| *SFRP4* | cg20019546 | 1stExon | Island | 0.465 | 0.377 | 0.088 | 1.234 | 1.30E-17 |
| *SFRP4* | cg21122375 | TSS1500 | S_Shore | 0.128 | 0.092 | 0.036 | 1.388 | 2.58E-14 |
| *SFRP4* | cg22826141 | TSS200 | S_Shore | 0.056 | 0.064 | -0.008 | 0.873 | 0.017017 |
| *SFRP4* | cg23169784 | 3'UTR | OpenSea | 0.885 | 0.942 | -0.057 | 0.939 | 3.35E-13 |
| *SFRP4* | cg23569180 | Body | N_Shore | 0.824 | 0.857 | -0.033 | 0.962 | 0.000885 |
| *SFRP4* | cg25783719 | 1stExon;5'UTR | Island | 0.033 | 0.026 | 0.007 | 1.273 | 0.01308 |
| *MOXD1* | cg01019028 | TSS200 | Island | 0.177 | 0.141 | 0.036 | 1.255 | 0.001906 |
| *MOXD1* | cg05235761 | Body | N_Shore | 0.276 | 0.118 | 0.158 | 2.343 | 8.59E-40 |
| *MOXD1* | cg05477953 | Body | N_Shelf | 0.669 | 0.924 | -0.254 | 0.725 | 2.86E-69 |
| *MOXD1* | cg07287384 | Body | OpenSea | 0.794 | 0.924 | -0.129 | 0.860 | 5.05E-48 |
| *MOXD1* | cg07570142 | 1stExon | Island | 0.320 | 0.144 | 0.176 | 2.217 | 3.28E-28 |
| *MOXD1* | cg08661899 | TSS200 | Island | 0.115 | 0.041 | 0.074 | 2.802 | 1.56E-19 |
| *MOXD1* | cg13603171 | TSS1500 | S_Shore | 0.341 | 0.284 | 0.057 | 1.200 | 1.12E-04 |
| *MOXD1* | cg13640626 | TSS200 | Island | 0.151 | 0.050 | 0.101 | 3.011 | 2.35E-24 |
| *MOXD1* | cg15594306 | TSS1500 | S_Shore | 0.285 | 0.318 | -0.033 | 0.896 | 5.81E-04 |
| *MOXD1* | cg16478774 | 1stExon | Island | 0.084 | 0.022 | 0.062 | 3.746 | 3.53E-21 |
| *MOXD1* | cg21294812 | TSS200 | Island | 0.201 | 0.083 | 0.118 | 2.424 | 1.74E-22 |
| *MOXD1* | cg24645868 | TSS1500 | S_Shore | 0.640 | 0.905 | -0.264 | 0.708 | 1.49E-55 |
| *MOXD1* | cg27495887 | Body | OpenSea | 0.829 | 0.917 | -0.088 | 0.904 | 1.45E-20 |
| *CXCL14* | cg01821923 | Body | Island | 0.230 | 0.135 | 0.095 | 1.701 | 3.00E-19 |
| *CXCL14* | cg07516956 | TSS200 | S_Shore | 0.140 | 0.033 | 0.107 | 4.269 | 1.13E-32 |
| *CXCL14* | cg08373187 | Body | N_Shelf | 0.236 | 0.371 | -0.135 | 0.637 | 4.18E-29 |
| *CXCL14* | cg10179196 | TSS200 | S_Shore | 0.284 | 0.321 | -0.037 | 0.884 | 0.00029 |
| *CXCL14* | cg17008288 | 3'UTR | OpenSea | 0.582 | 0.817 | -0.234 | 0.713 | 3.03E-56 |
| *CXCL14* | cg18995088 | 1stExon;5'UTR | Island | 0.146 | 0.079 | 0.067 | 1.847 | 8.89E-07 |
| *CXCL14* | cg23510026 | TSS1500 | S_Shore | 0.597 | 0.846 | -0.249 | 0.706 | 3.27E-47 |
| *CXCL14* | cg25011337 | TSS200 | S_Shore | 0.136 | 0.116 | 0.020 | 1.174 | 0.031109 |
| *CXCL14* | cg26393379 | Body | N_Shore | 0.294 | 0.376 | -0.082 | 0.781 | 7.30E-09 |
| *CXCL14* | cg26525592 | TSS1500 | S_Shore | 0.379 | 0.562 | -0.183 | 0.674 | 5.71E-23 |
| *CXCL14* | cg27090201 | 1stExon;5'UTR | Island | 0.087 | 0.023 | 0.065 | 3.808 | 2.59E-14 |
| *STMN2* | cg00347369 | TSS200 | N_Shore | 0.244 | 0.192 | 0.052 | 1.270 | 3.76E-07 |
| *STMN2* | cg00397910 | TSS1500 | N_Shore | 0.375 | 0.528 | -0.152 | 0.711 | 1.12E-30 |
| *STMN2* | cg00398130 | Body | Island | 0.394 | 0.259 | 0.135 | 1.521 | 5.30E-16 |
| *STMN2* | cg01691768 | Body | Island | 0.183 | 0.124 | 0.059 | 1.480 | 9.04E-17 |
| *STMN2* | cg01774159 | Body | Island | 0.205 | 0.073 | 0.132 | 2.815 | 8.39E-34 |
| *STMN2* | cg08496775 | TSS200 | N_Shore | 0.268 | 0.329 | -0.062 | 0.813 | 1.35E-08 |
| *STMN2* | cg09071889 | 1stExon;5'UTR | N_Shore | 0.338 | 0.182 | 0.156 | 1.860 | 9.88E-27 |
| *STMN2* | cg13042329 | Body | S_Shelf | 0.499 | 0.775 | -0.276 | 0.643 | 3.31E-36 |
| *STMN2* | cg14284469 | Body | Island | 0.230 | 0.111 | 0.118 | 2.060 | 9.74E-26 |
| *STMN2* | cg15827214 | TSS1500 | N_Shore | 0.825 | 0.916 | -0.091 | 0.901 | 5.01E-21 |
| *STMN2* | cg16840523 | Body | Island | 0.131 | 0.071 | 0.060 | 1.853 | 1.61E-15 |
| *STMN2* | cg17337106 | 3'UTR | OpenSea | 0.774 | 0.939 | -0.166 | 0.823 | 2.29E-44 |
| *STMN2* | cg20046330 | TSS200 | N_Shore | 0.323 | 0.260 | 0.063 | 1.243 | 1.16E-09 |
| *STMN2* | cg22750155 | Body | Island | 0.196 | 0.029 | 0.167 | 6.868 | 3.77E-45 |
| *STMN2* | cg23326689 | Body | N_Shore | 0.354 | 0.287 | 0.066 | 1.231 | 7.41E-06 |
| *STMN2* | cg26577017 | TSS200 | N_Shore | 0.318 | 0.495 | -0.177 | 0.642 | 1.58E-28 |
| *STMN2* | cg26683058 | TSS1500 | N_Shore | 0.772 | 0.847 | -0.075 | 0.912 | 7.88E-07 |
| *STMN2* | cg27326514 | Body | S_Shore | 0.618 | 0.705 | -0.087 | 0.877 | 1.79E-07 |
| *FCN3* | cg01719260 | TSS1500 | OpenSea | 0.777 | 0.863 | -0.086 | 0.901 | 2.93E-19 |
| *FCN3* | cg02365086 | Body | OpenSea | 0.485 | 0.735 | -0.250 | 0.660 | 1.58E-49 |
| *FCN3* | cg11529819 | 3'UTR | S_Shelf | 0.478 | 0.551 | -0.073 | 0.867 | 8.29E-13 |
| *FCN3* | cg13082816 | TSS1500 | OpenSea | 0.603 | 0.747 | -0.143 | 0.808 | 3.51E-32 |
| *FCN3* | cg19979773 | TSS1500 | OpenSea | 0.812 | 0.876 | -0.064 | 0.926 | 7.23E-15 |
| *FCN3* | cg20775044 | TSS1500 | OpenSea | 0.892 | 0.944 | -0.052 | 0.945 | 9.58E-08 |
| *FCN3* | cg22679725 | TSS1500 | OpenSea | 0.827 | 0.893 | -0.066 | 0.926 | 2.67E-15 |
| *COMP* | cg02146857 | Body | Island | 0.275 | 0.228 | 0.047 | 1.207 | 8.04E-09 |
| *COMP* | cg02201071 | Body | Island | 0.177 | 0.144 | 0.033 | 1.231 | 0.000751 |
| *COMP* | cg02624809 | Body | Island | 0.188 | 0.125 | 0.063 | 1.505 | 7.04E-11 |
| *COMP* | cg03767475 | TSS1500 | S_Shore | 0.535 | 0.791 | -0.257 | 0.676 | 2.25E-54 |
| *COMP* | cg04273661 | Body | Island | 0.681 | 0.614 | 0.068 | 1.110 | 4.29E-10 |
| *COMP* | cg04726439 | Body | Island | 0.525 | 0.405 | 0.120 | 1.296 | 4.38E-24 |
| *COMP* | cg07856714 | TSS200 | Island | 0.141 | 0.114 | 0.028 | 1.244 | 0.001422 |
| *COMP* | cg09949775 | 1stExon;5'UTR | Island | 0.420 | 0.225 | 0.195 | 1.867 | 9.44E-27 |
| *COMP* | cg09980058 | Body | Island | 0.470 | 0.131 | 0.339 | 3.588 | 1.87E-68 |
| *COMP* | cg11086760 | Body | Island | 0.115 | 0.099 | 0.016 | 1.159 | 0.031654 |
| *COMP* | cg11609001 | Body | Island | 0.222 | 0.069 | 0.153 | 3.223 | 6.65E-27 |
| *COMP* | cg12020549 | Body | Island | 0.067 | 0.034 | 0.033 | 1.955 | 0.007195 |
| *COMP* | cg15424739 | Body | Island | 0.211 | 0.035 | 0.176 | 5.970 | 7.00E-34 |
| *COMP* | cg15986030 | TSS200 | Island | 0.350 | 0.259 | 0.091 | 1.350 | 1.02E-14 |
| *COMP* | cg19480198 | Body | Island | 0.476 | 0.274 | 0.202 | 1.735 | 1.42E-26 |
| *COMP* | cg19485202 | 1stExon;5'UTR | Island | 0.426 | 0.208 | 0.218 | 2.051 | 9.15E-31 |
| *COMP* | cg19907305 | TSS200 | Island | 0.395 | 0.253 | 0.142 | 1.561 | 1.39E-21 |
| *COMP* | cg20185461 | Body | Island | 0.541 | 0.333 | 0.207 | 1.622 | 8.27E-43 |
| *COMP* | cg22865824 | TSS1500 | S_Shore | 0.461 | 0.698 | -0.236 | 0.661 | 2.05E-58 |
| *COMP* | cg24924091 | Body | Island | 0.166 | 0.038 | 0.128 | 4.342 | 5.71E-23 |
| *COMP* | cg25497529 | Body | Island | 0.158 | 0.057 | 0.101 | 2.786 | 4.92E-23 |
| *COMP* | cg26669806 | Body | Island | 0.376 | 0.120 | 0.256 | 3.135 | 1.07E-55 |
| *LAMA2* | cg00334550 | Body | N_Shore | 0.582 | 0.903 | -0.322 | 0.644 | 1.83E-71 |
| *LAMA2* | cg00991202 | Body | OpenSea | 0.552 | 0.851 | -0.299 | 0.648 | 7.54E-72 |
| *LAMA2* | cg01850783 | Body | OpenSea | 0.103 | 0.085 | 0.019 | 1.221 | 0.033777 |
| *LAMA2* | cg01916115 | 1stExon;5'UTR | OpenSea | 0.210 | 0.140 | 0.069 | 1.493 | 6.64E-12 |
| *LAMA2* | cg02873997 | Body | OpenSea | 0.722 | 0.976 | -0.253 | 0.740 | 1.01E-47 |
| *LAMA2* | cg03658275 | Body | OpenSea | 0.395 | 0.737 | -0.342 | 0.536 | 1.46E-51 |
| *LAMA2* | cg04831510 | Body | N_Shore | 0.650 | 0.926 | -0.276 | 0.702 | 2.32E-69 |
| *LAMA2* | cg05008344 | Body | OpenSea | 0.501 | 0.774 | -0.273 | 0.647 | 5.43E-41 |
| *LAMA2* | cg05140624 | Body | S_Shore | 0.396 | 0.611 | -0.215 | 0.648 | 1.68E-37 |
| *LAMA2* | cg09142843 | Body | OpenSea | 0.510 | 0.823 | -0.313 | 0.619 | 4.93E-65 |
| *LAMA2* | cg09241097 | Body | OpenSea | 0.790 | 0.873 | -0.083 | 0.905 | 1.83E-20 |
| *LAMA2* | cg10642200 | Body | OpenSea | 0.651 | 0.926 | -0.276 | 0.702 | 1.72E-77 |
| *LAMA2* | cg12802286 | Body | OpenSea | 0.686 | 0.919 | -0.233 | 0.746 | 1.75E-51 |
| *LAMA2* | cg14315006 | Body | OpenSea | 0.701 | 0.897 | -0.196 | 0.781 | 9.22E-26 |
| *LAMA2* | cg14919130 | Body | N_Shore | 0.485 | 0.896 | -0.411 | 0.541 | 5.84E-88 |
| *LAMA2* | cg15801019 | TSS1500 | OpenSea | 0.270 | 0.331 | -0.061 | 0.816 | 2.09E-05 |
| *LAMA2* | cg16120501 | Body | OpenSea | 0.733 | 0.981 | -0.248 | 0.747 | 2.68E-47 |
| *LAMA2* | cg17255005 | Body | OpenSea | 0.396 | 0.630 | -0.233 | 0.629 | 5.60E-32 |
| *LAMA2* | cg17422019 | Body | OpenSea | 0.394 | 0.754 | -0.360 | 0.522 | 1.38E-65 |
| *LAMA2* | cg19358568 | Body | OpenSea | 0.611 | 0.941 | -0.330 | 0.649 | 1.22E-68 |
| *LAMA2* | cg19625507 | Body | OpenSea | 0.570 | 0.689 | -0.118 | 0.828 | 1.86E-28 |
| *LAMA2* | cg20237610 | TSS1500 | OpenSea | 0.340 | 0.396 | -0.056 | 0.858 | 0.000129 |
| *LAMA2* | cg20640433 | TSS200 | OpenSea | 0.225 | 0.155 | 0.070 | 1.449 | 2.13E-07 |
| *LAMA2* | cg21537798 | Body | OpenSea | 0.643 | 0.915 | -0.272 | 0.703 | 2.13E-66 |
| *LAMA2* | cg23143313 | Body | Island | 0.409 | 0.441 | -0.032 | 0.927 | 0.0062 |
| *LAMA2* | cg23621912 | Body | OpenSea | 0.265 | 0.527 | -0.261 | 0.504 | 1.96E-50 |
| *LAMA2* | cg24493834 | Body | N_Shore | 0.292 | 0.422 | -0.130 | 0.692 | 1.37E-24 |
| *LAMA2* | cg24636327 | Body | OpenSea | 0.518 | 0.833 | -0.315 | 0.622 | 3.21E-66 |
| *LAMA2* | cg25608547 | Body | N_Shore | 0.500 | 0.715 | -0.214 | 0.700 | 2.40E-25 |
| *LAMA2* | cg26926076 | Body | OpenSea | 0.066 | 0.036 | 0.030 | 1.848 | 2.89E-08 |
| *LAMA2* | cg27120934 | Body | OpenSea | 0.074 | 0.286 | -0.212 | 0.259 | 4.03E-28 |
| *LAMA2* | cg27192990 | Body | OpenSea | 0.700 | 0.793 | -0.093 | 0.882 | 4.97E-23 |
| *LAMA2* | cg27439042 | Body | OpenSea | 0.528 | 0.892 | -0.364 | 0.592 | 1.39E-64 |
| *CPA3* | cg03020424 | TSS1500 | OpenSea | 0.644 | 0.855 | -0.211 | 0.753 | 1.82E-44 |
| *CPA3* | cg13424229 | TSS1500 | OpenSea | 0.701 | 0.888 | -0.187 | 0.789 | 1.66E-39 |
| *CPA3* | cg18516150 | 3'UTR | OpenSea | 0.345 | 0.615 | -0.270 | 0.561 | 2.01E-55 |
| *CPA3* | cg24290574 | 1stExon | OpenSea | 0.514 | 0.711 | -0.197 | 0.723 | 1.42E-29 |
| *NPY1R* | cg03743432 | 5'UTR;1stExon | S_Shore | 0.232 | 0.169 | 0.063 | 1.373 | 0.000378 |
| *NPY1R* | cg05445244 | 5'UTR | N_Shore | 0.497 | 0.647 | -0.150 | 0.768 | 6.20E-19 |
| *NPY1R* | cg06734271 | TSS1500 | S_Shore | 0.071 | 0.051 | 0.019 | 1.372 | 1.47E-05 |
| *NPY1R* | cg07451928 | 5'UTR | N_Shelf | 0.595 | 0.838 | -0.243 | 0.710 | 1.06E-32 |
| *NPY1R* | cg08767627 | 5'UTR | N_Shore | 0.693 | 0.668 | 0.025 | 1.038 | 0.04055 |
| *NPY1R* | cg09082903 | 5'UTR | Island | 0.284 | 0.062 | 0.222 | 4.606 | 3.71E-38 |
| *NPY1R* | cg09440095 | TSS200 | S_Shore | 0.102 | 0.071 | 0.031 | 1.439 | 6.49E-07 |
| *NPY1R* | cg15896624 | Body | OpenSea | 0.434 | 0.738 | -0.304 | 0.588 | 5.78E-47 |
| *NPY1R* | cg16358044 | 3'UTR | OpenSea | 0.403 | 0.653 | -0.250 | 0.617 | 2.37E-44 |
| *NPY1R* | cg17500103 | TSS1500 | S_Shore | 0.064 | 0.077 | -0.013 | 0.835 | 0.011445 |
| *NPY1R* | cg19908812 | 5'UTR | Island | 0.382 | 0.154 | 0.228 | 2.484 | 1.45E-40 |
| *NPY1R* | cg22714094 | TSS200 | S_Shore | 0.112 | 0.039 | 0.073 | 2.861 | 5.27E-20 |
| *NPY1R* | cg24157598 | TSS1500 | S_Shore | 0.821 | 0.883 | -0.062 | 0.930 | 3.41E-17 |
| *NPY1R* | cg24535910 | 5'UTR | Island | 0.255 | 0.153 | 0.101 | 1.661 | 2.42E-16 |

*Note*: The information was from DNMIVD and SurvivalMeth databases.
